# Expression of myeloid Src-family kinases is associated with poor prognosis in AML and influences Flt3-ITD kinase inhibitor acquired resistance

**DOI:** 10.1371/journal.pone.0225887

**Published:** 2019-12-02

**Authors:** Ravi K. Patel, Mark C. Weir, Kexin Shen, Daniel Snyder, Vaughn S. Cooper, Thomas E. Smithgall

**Affiliations:** Department of Microbiology and Molecular Genetics, University of Pittsburgh School of Medicine, Pittsburgh, Pennsylvania, United States of America; Pennsylvania State University, UNITED STATES

## Abstract

Unregulated protein-tyrosine kinase signaling is a common feature of AML, often involving mutations in Flt3 and overexpression of myeloid Src-family kinases (Hck, Fgr, Lyn). Here we show that high-level expression of these Src kinases predicts poor survival in a large cohort of AML patients. To test the therapeutic benefit of Flt3 and Src-family kinase inhibition, we used the pyrrolopyrimidine kinase inhibitor A-419259. This compound potently inhibits Hck, Fgr, and Lyn as well as Flt3 bearing an activating internal tandem duplication (ITD). Flt3-ITD expression sensitized human TF-1 myeloid cells to growth arrest by A-419259, supporting direct action on the Flt3-ITD kinase domain. Cells transformed with the Flt3-ITD mutants D835Y and F691L were resistant to A-419259, while co-expression of Hck or Fgr restored inhibitor sensitivity to Flt3-ITD D835Y. Conversely, Hck and Fgr mutants with engineered A-419259 resistance mutations decreased sensitivity of TF-1/Flt3-ITD cells. To investigate *de novo* resistance mechanisms, A-419259-resistant Flt3-ITD^+^ AML cell populations were derived via long-term dose escalation. Whole exome sequencing identified a distinct Flt3-ITD kinase domain mutation (N676S/T) among all A-419259 target kinases in each of six independent resistant cell populations. These studies show that Hck and Fgr expression influences inhibitor sensitivity and the pathway to acquired resistance in Flt3-ITD^+^ AML.

## Introduction

Acute myeloid leukemia (AML) is characterized by unchecked expansion of undifferentiated myeloid blast cells that ultimately take over the bone marrow, resulting in suppression of normal hematopoiesis [1]. Currently, AML patients have only a 40% five-year survival rate and most are limited to a chemotherapy regimen that has changed little over the past 45 years [2]. While multiple genetic changes are associated with AML, upregulation of protein-tyrosine kinase signaling is a common theme that offers an opportunity for targeted therapy. One important example involves the FMS-like tyrosine kinase 3 (Flt3) receptor tyrosine kinase, which is often over-expressed [3] or mutated in AML [4]. Flt3 and its associated ligand regulate normal hematopoiesis and are expressed by progenitor cells of the myeloid and lymphoid lineages [5]. Mutations in Flt3 result in ligand-independent kinase activity and leukemogenesis [6], defining Flt3 as a classic proto-oncogene in AML. Activating Flt3 mutations occur as either internal tandem duplication (ITD) events in the cytosolic juxtamembrane region or as point mutations in the tyrosine kinase domain [7,8]. Flt3-ITD mutations are more common and associated with a worse prognosis [9,10].

The identification of Flt3-ITD as a common driver mutation in AML led to the development of Flt3 kinase inhibitors as an approach to precision therapy. Flt3 inhibitors have had some success in clinical trials although low response rates and acquired resistance remain as vexing problems [11], even for the recently FDA-approved Flt3 inhibitor midostaurin [12,13]. Most patients develop resistance to Flt3 inhibitors through mutations in the kinase domain that affect inhibitor binding but not kinase activity [14,15]. For example, midostaurin resistance can arise from substitution of kinase domain residue Asn676, which forms a network of hydrogen bonds to stabilize inhibitor binding [16]. Quizartinib is another Flt3 inhibitor with clinical promise for AML [17]. While quizartinib is a potent and highly selective Flt3 inhibitor, single kinase domain point mutations can confer complete resistance, including F691L, D835Y and Y842C [15]. The rapid evolution of Flt3 kinase inhibitor resistance underscores the need for strategies that limit emergence of Flt3 mutants that acutely evade treatment and thus minimize the prospect of recurrent disease.

One promising approach to suppress the emergence of inhibitor resistance is to use compounds that target not only Flt3, but also other AML-associated tyrosine kinases. Myeloid Src-family kinases, including Hck, Lyn and Fgr, are frequently over-expressed in AML leukemic stem cells [18,19] and represent attractive targets in this regard. Our group has recently shown that Hck, Lyn and Fgr are commonly overexpressed in bone marrow cells from AML patients, consistent with these findings [20]. In addition, AML stem cells have much higher Src-family kinase activity than normal hematopoietic stem cells and myeloid cells [18,19]. High expression and kinase activity suggest that selective inhibitors of Hck, Lyn and Fgr will reduce AML cell viability. This idea is reinforced by RNAi-knockdown studies of these Src-family members, where reduced kinase expression correlates with growth arrest and increased apoptosis in primary AML cells [19].

Strong evidence specifically implicates Hck in AML. Saito *et al*. found Hck to be overexpressed in leukemic stem cells from AML patients who had relapsed from chemotherapy [21] and showed that shRNA-mediated knockdown of Hck arrested growth of leukemic blasts [22]. This group went on to show that the pyrrolopyrimidine Src-family kinase inhibitor A-419259, referred to as RK-20449 in Saito *et al*. [22], completely eliminates chemotherapy-resistant AML patient xenografts in mice. Subsequent work has shown that A-419259 is also an inhibitor of Flt3 kinase activity in vitro, suggesting that its activity against multiple AML-associated tyrosine kinases may account for its efficacy against primary patient cells in the mouse xenograft model [23]. In addition to Hck, recent evidence has identified Fgr as an oncogene and a therapeutic target in AML. In contrast to Hck, expression of wild-type Fgr without mutation induces oncogenic transformation of rodent fibroblasts in vitro, and reduces the cytokine dependence of a human myeloid leukemia cell line in colony-forming assays [24]. In addition, an *N*-phenylbenzamide kinase inhibitor, TL02-59, potently inhibits Fgr kinase activity in vitro and in vivo. This compound is most active against bone marrow cells from AML patients that express high levels of Fgr as well as Hck [20].

In the present study, we combined the use of kinases with engineered inhibitor resistance mutations and *in vitro* selection of resistance to determine the roles of Flt3, Hck and Fgr in the sensitivity of AML cells to A-419259 treatment. Human myeloid leukemia cells transformed with Flt3-ITD acquired remarkable sensitivity to A-419259 in the presence or absence of Hck or Fgr. Cells transformed with mutants of Flt3-ITD known to cause resistance to other kinase inhibitors (D835Y and F691L) completely lost their A-419259 sensitivity, validating Flt3-ITD as a direct target for this inhibitor in cells. When Hck or Fgr were co-expressed with the Flt3-ITD D835Y mutant, A-419259 sensitivity was partially restored. Conversely, co-expression of wild-type Flt3-ITD with engineered A-419259-resistant mutants of Hck or Fgr resulted in partial resistance to A-419259. In an unbiased approach, we experimentally evolved A-419259-resistant populations of Flt3-ITD^+^ AML cell lines (which also express endogenous active Hck and Fgr) through gradual dose escalation, which required many months. Subsequent whole exome sequence analysis revealed a distinct resistance mutation at a single position in the Flt3 kinase domain (N676 only), but not in Hck, Fgr, or any other A-419259 target kinases identified by KINOMEscan analysis. Together, our results show that Hck and Fgr expression influences both A-419259 sensitivity and the pathway to acquired resistance to this compound in Flt3-ITD^+^ AML.

## Materials and methods

### Kinase inhibitors

A-419259 was obtained from Sigma-Aldrich. Quizartinib (AC220) was purchased from LC laboratories. TL02-59 was custom synthesized by A Chemtek, Inc. The Syk inhibitor, PRT062607, was purchased from Selleckchem.

### KINOMEscan analysis of A-419259 target specificity

The target kinase specificity of A-419259 was profiled using the KINOMEscan scanMAX service from Eurofins/DiscoverX as previously described [25]. KINOMEscan is a competitive binding assay in which a DNA-tagged kinase is incubated with a compound of interest in the presence of immobilized, non-selective ATP analogs. Kinase retention to the immobilized ligand is then measured using quantitative real-time PCR for each kinase-specific DNA barcode. Results are reported as the percent of each kinase that remains bound to the immobilized ATP analog. Data were visualized using the TREEspot^™^ profile visualization tool version 5.0 (Eurofins) which shows the interacting kinases on a circular dendrogram representing the entire human kinome.

### Recombinant protein kinases

Recombinant purified Flt3 wild-type, Flt3-ITD and Flt3-D835Y kinase domains were purchased from ThermoFisher. Recombinant near-full-length Hck, Lyn and Fgr were co-expressed with Csk and PTP1B in BL21Star (DE3) *E*. *coli* in 1 L terrific broth. Once the culture reached an OD_600_ of 1.0, protein expression was induced with 0.5 mM IPTG for 16 h at 16 °C. The bacterial cell pellet was lysed using a Microfluidics M-110P microfluidizer and clarified by ultracentrifugation. Recombinant kinases were purified through sequential HisTrap HP, HiTrap Blue and HiLoad 26/600 Superdex columns using an ÄKTA Explorer automated chromatography system (GE Healthcare Life Sciences).

### Z’-LYTE in vitro kinase assay

The Z’Lyte in vitro kinase assay (Life Technologies) is described in detail elsewhere [26,27]. Assays were performed in quadruplicate in 384-well low volume, non-binding, black polystyrene microplates (Corning) according to the manufacturer’s instructions. Briefly, this assay measures phosphorylation of the Tyr2 peptide substrate which is tagged with coumarin and fluorescein on its N- and C-termini, respectively, to form a FRET pair. After the kinase reaction, a development step involves site-specific proteolytic cleavage of the unphosphorylated but not the phosphorylated peptide. Peptide cleavage results in loss of the FRET signal. Kinase reactions were run in 50 mM HEPES, pH 7.5, 10 mM MgCl_2_, 1 mM EGTA, and 0.01% BRIJ-35 (buffer supplied by the manufacturer). Kinase reactions were initiated by the addition of ATP at the K_m_ for each kinase and Tyr2 peptide substrate (1 μM). Following incubation for 1 h at ambient temperature, reactions were quenched by addition of the development protease, and coumarin and fluorescein fluorescence were measured 1 h later on a Molecular Devices SpectraMax M5 plate reader. Data are expressed as a ratio of the coumarin (445 nm) to fluorescein FRET (520 nM) emissions normalized to signals observed in the absence of ATP (negative control) and with a positive control peptide in the absence of kinase (Tyr2 that is 100% phosphorylated). For experiments with inhibitors, compounds were preincubated with kinase for 30 min prior to initiation of the reaction by the addition of ATP.

### Cell culture

TF-1 and MV4-11 cells were obtained from the American Type Culture Collection (ATCC) and cultured in RPMI 1640 medium supplemented with 10% fetal bovine serum (FBS), 100 units/ml of penicillin, 100 μg/ml of streptomycin sulfate, and 0.25 μg/ml of amphotericin B (Antibiotic-Antimycotic; Gibco/ThermoFisher). Parental TF-1 cells also require recombinant human GM-CSF (1 ng/mL; ThermoFisher). MOLM13 and MOLM14 cells were obtained from Leibniz Institute Deutsche Sammlung von Mikroorganismen (DSMZ) and grown in RPMI 1640 medium containing 20% FBS and Antibiotic-Antimycotic. Human 293T cells were obtained from ATCC and cultured in Dulbecco's modified Eagle's medium (DMEM) containing 10% FBS and Antibiotic-Antimycotic.

### Generation of TF-1 cell lines stably expressing Flt3, Hck, or Fgr

Full-length cDNA clones of each kinase were subcloned into the retroviral expression vectors pMSCV-neo or pMSCV-puro (Clontech). High-titer retroviral stocks were produced in 293T cells co-transfected with each pMSCV construct and an amphotropic packaging vector. TF-1 cells (10^6^) were resuspended in 5.0 mL of undiluted viral supernatant and centrifuged at 1,000 × g for 4 h at 18 °C in the presence of 4 μg/mL Polybrene (Sigma-Aldrich) to enhance viral transduction. Forty-eight hours after infection, the cells began a two-week selection period with 400 μg/ml G-418 (neo vectors) or 3 μg/mL puromycin (puro vectors). Following selection, cells were maintained with 200 μg/ml G418 or 1 μg/mL puromycin. For double transduction experiments, TF-1 cells were first infected with the pMSCV-neo-Flt3-ITD virus, selected with G418, followed by the pMSCV-puro virus carrying Hck or Fgr and puromycin selection. All TF-1 cell populations that expressed Flt3-ITD (including inhibitor-resistant mutants) proliferated in a GM-CSF-independent manner, and were therefore subcultured in the absence of GM-CSF.

### Cell viability assay

Cells were seeded at 10^5^ per mL in the presence or absence of inhibitors with DMSO as carrier solvent (0.1% final). Cell viability was assessed using the CellTiter-Blue reagent according to the manufacturer’s instructions (Promega). Fluorescence intensity, which correlates directly with viable cell number, was measured using a SpectraMax M5 microplate reader. Each experiment included three technical replicates per condition, and each experiment was repeated at least three times.

### Immunoprecipitation and immunoblotting

Cells (3 x 10^6^ per 5 mL medium) were cultured with inhibitors or the DMSO carrier solvent alone as control (final DMSO concentration of 0.1% in each case) for 16 h. Cells were then lysed in RIPA buffer (50 mM Tris-HCl, pH 7.4, 150 mM NaCl, 1 mM EDTA, 1% Triton X-100, 0.1% SDS, 1% sodium deoxycholate) supplemented with 2.5 mM sodium orthovanadate, 25 mM sodium fluoride, 5 units/mL Benzonase (Novagen), and a protease inhibitor cocktail (cOmplete EDTA-Free tablets; Sigma). Protein concentrations in the lysates were determined using Protein Assay Dye concentrate (BioRad).

Kinases were immunoprecipitated using anti-Flt3 (CST #3462), anti-Hck (CST #14643S), or anti-Fgr (CST #2755S) antibodies (Cell Signaling Technologies). Each immunoprecipitation reaction contained 1 mg lysate protein, 2 μg antibody, and 20 μL of protein G-Sepharose beads (Invitrogen) in RIPA buffer with supplements as described above. Immunoprecipitation reactions were incubated overnight at 4 °C. Immunoprecipitates were collected by micro-centrifugation, washed two times by resuspension in 1.0 mL RIPA buffer, separated by SDS-PAGE and transferred to nitrocellulose membranes. Flt3 was blotted with anti-Flt3 (Cell Signaling Technologies #3462) and anti-phosphotyrosine (pY99; Santa Cruz sc-7020) antibodies. Hck was blotted with anti-Hck (Cell Signaling Technologies #14643S) and anti-phospho-Src (pTyr416) clone 9A6 (EMD Millipore) antibodies. Fgr was blotted with anti-Fgr (Cell Signaling Technologies #2755S) and anti-phospho-Src (pTyr416) clone 9A6. Secondary antibodies included anti-mouse or anti-rabbit IgG conjugated to 680 nM and 800 nM fluorophores, respectively (LI-COR). Blots were scanned using a LI-COR Odyssey imager, and signal intensities were quantified using the Image Studio Lite software. Data were plotted as ratios of the phosphoprotein to total protein signals and fit by non-linear regression analysis to determine the IC_50_ values.

### Experimental evolution of A-419259-resistant populations of AML cell lines

Populations of MV4-11, MOLM13 and MOLM14 cells with de novo resistance to A-419259 were initiated by culturing 10^6^ cells in 5.0 mL of medium containing A-419259 at a starting concentration of 10 nM. Viability of each cell population was measured three times per week using the CellTiter-Blue assay. Once cell viability crossed a threshold of 4000 RFU over background in this assay, 10^6^ cells were subcultured into 1.0 mL fresh medium containing 50% more compound (i.e. cells growing in 10 nM would be passed into 15 nM). Cells were sub-cultured in this way until outgrowth was observed in the presence of 1 μM A-419259.

### Exome sequencing and analysis

Genomic DNA was prepared for sequencing using the Illumina TruSeq Rapid Exome kit, and 150 bp paired-end sequencing was performed with a mid-output flowcell (Illumina FC-404-2003) on an Illumina NextSeq-500 sequencer. Data analysis was performed at the University of Pittsburgh Center for Research Computing using the Genome analysis Toolkit (GATK) best practices [28]. Contaminating 5’ and 3’ adapter sequences were removed with Trimmomatic version 0.33 [29], resulting in ~135 bp paired-end reads with greater than 30-fold average coverage of the exome for each sample. Sequence reads were aligned to human reference genome hg37 with Burrows-Wheeler aligner maximal exact matches (BWA-MEM) algorithm version 0.7.15. Samtools version 1.3.1 was used to convert the sam file to bam file format. Duplicates were removed and base scores were recalibrated with Picard Mark Duplicates version 2.11.0 [http://broadinstitute.github.io/picard]. Variant calling files (VCFs) were generated using GATK version 3.8.1 Haplotypecaller [30]. SnpEff version 4.3 was used to annotate the VCF [31]. Finally, Cancer-specific High-throughput Annotation of Somatic Mutations (CHASM) was used to rank the variants [32]. All software code is freely available at this link: https://github.com/RaviKPatel-PhD/Hck-and-Fgr-Regulate-Sensitivity-of-Flt3-ITD-AML. Raw sequencing data are available via the NCBI sequence read archive (SRA).

### RNA Isolation, cDNA preparation, qPCR

Total RNA was isolated from cells using the RNeasy Plus Mini Kit (Qiagen). cDNA was prepared from total RNA using the RETROscript kit (ThermoFisher/Invitrogen). Real-time quantitative RT-PCR assays were performed on total RNA using SYBR Green detection and gene-specific QuantiTect primers (Qiagen) on an Applied Biosystems StepONE plus real-time PCR instrument.

### Statistical analysis

All statistical analyses, including non-linear regression analyses, Student’s t-tests, and ANOVA were performed with the Prism software package, version 8.0 (GraphPad Software, Inc.).

## Results

### Myeloid Src-family kinase expression predicts patient survival in AML

**Fig 1:**
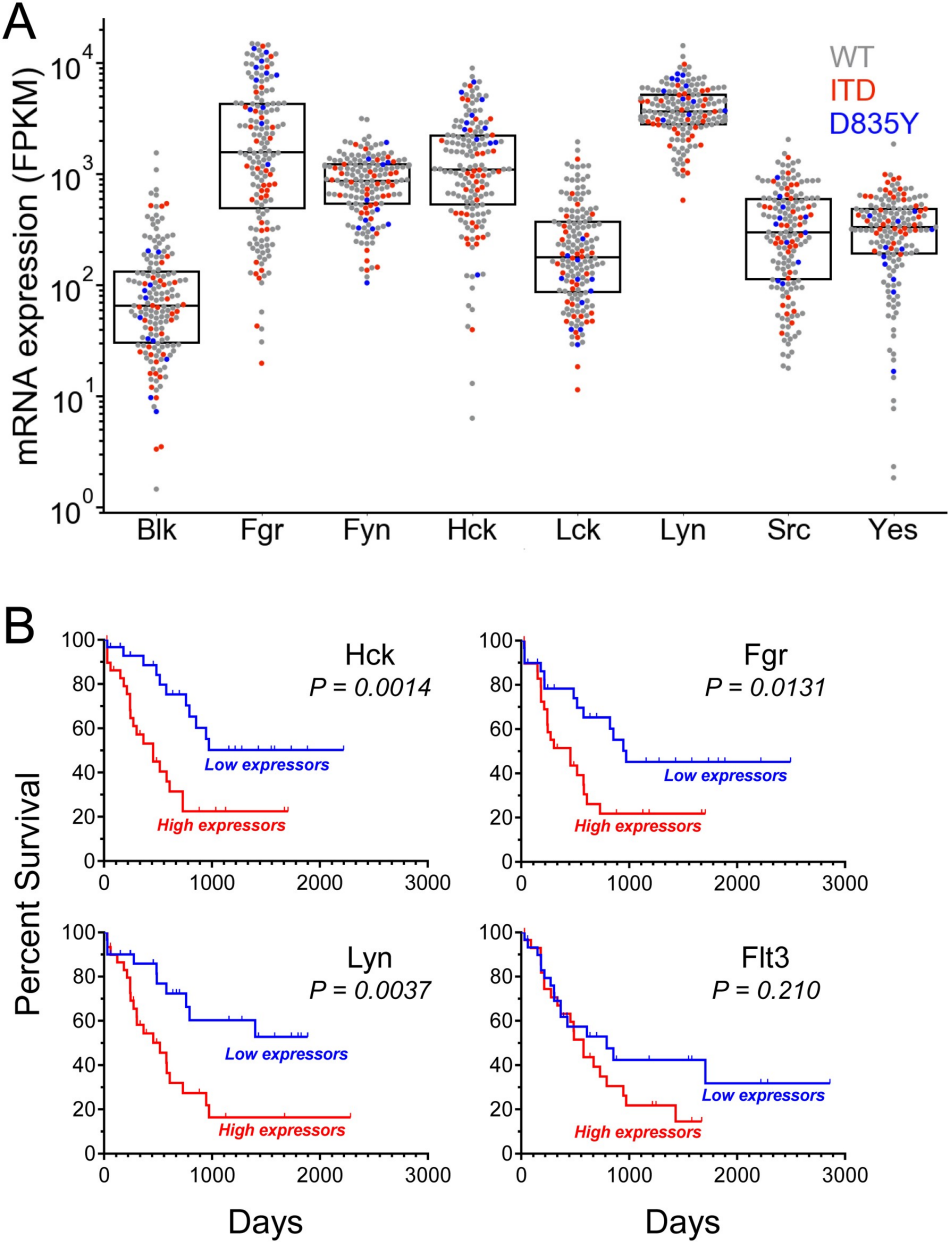
Expression profiles of Src-family kinases in AML and their relationship to patient survival. (**A**) Gene expression of the eight mammalian Src-family members in samples from all AML patients in The Cancer Genome Atlas (TCGA) cohort (n = 163). Transcript data are shown as the number of kinase cDNA fragments per kilobase of transcript per million mapped reads (FPKM). Dots represent individual patient expression data, with the dot color representing Flt3 mutational status (grey, wild type; red, ITD; blue, D835Y). The boxplot shows the mean and quartile (25–75%) expression values for each kinase. Expression values of each Src-family member were compared to that of the B cell kinase Blk as reference control. One-way ANOVA showed significant differences in Fgr, Hck, Lyn, and Fyn levels relative to Blk (p < 0.0001), while Src, Lck and Yes expression levels were not significantly different. (**B**) Kaplan-Meier survival analysis for AML patients with the highest (top 20%; red) and lowest (bottom 20%; blue) mRNA levels for FGR, HCK, LYN and FLT3 (30 patients per group from 150 cases where survival data was available). The survival difference between patients with high vs. low expression is significant for the Src-family kinases but not FLT3 (P value shown from Mantel-Cox test).

Knockdown of Hck, Lyn and Fgr expression has been shown to decrease proliferation of patient AML cells, suggesting that the activity of these kinases is essential for disease progression [19]. However, the extent to which these kinases are expressed in AML as well as their relationship to disease outcome has not been analyzed in detail. To address these important issues, we first examined mRNA expression data in 163 AML patient samples publicly available from the Cancer Genome Atlas (TCGA) database. For comparative purposes, we used expression of Blk as a reference control because this Src-family member is expressed almost exclusively in B cells and only at very low levels in myeloid cells. One-way ANOVA shows a highly significant difference between Fgr, Fyn, Hck and Lyn expression levels relative to Blk (p < 0.0001), while Src, Yes and Lck are not significantly different (Fig 1A). Hck and Fgr showed a broad distribution of expression across the samples, while the levels of Lyn were more tightly clustered. Furthermore, pairwise analysis of Hck, Fgr and Lyn transcript levels revealed that expression of these kinases is highly correlated within individual patients (Supporting Information, S1A Fig). From a drug development point of view, Fgr and Hck are almost exclusively expressed in cells of myeloid lineage, while Fyn and Lyn have more broad-based expression patterns. Thus focusing inhibitor development on Hck and Fgr for AML may have the lowest risk of immunosuppression or other off-target toxicities.

We also investigated whether the distribution of Hck, Fgr, and Lyn expression levels from the TCGA AML data set were associated with other common AML mutations, including FLT3, NPM1, IDH1/2, DNM3A, RUNX1, p53, NRAS, CEBPA, WT1, and PTPN11 (S1B Fig). Hck, Fgr, and Lyn transcript distributions were very similar in each mutational subset when compared to all of the samples, suggesting that myeloid Src-family kinase expression does not segregate with a particular mutation. One exception is the subset of samples of Flt3-D835Y mutation, which showed significantly higher expression of Hck and Fgr compared to all AML samples. However, this difference may reflect the relatively small number of samples in the Flt3-D835Y group.

To determine whether myeloid Src-family kinase expression correlated with prognosis, we performed Kaplan-Meier analysis on the 150 AML patient samples from TCGA where survival data was available. Hck, Fgr and Lyn expression were all strongly predictive of patient prognosis. The 20% of patients with the highest levels of Hck, Lyn or Fgr expression showed the worst survival outcomes compared to the 20% with the lowest expression (Fig 1B). In contrast, expression levels of Flt3 did not correlate with significant differences in survival. Furthermore, Hck, Fgr and Lyn are most highly expressed in AML compared to other cancer types, with the exception of diffuse large B-cell lymphoma (DBLC; Supporting Information S2 Fig). Taken together, these results provide strong support for the idea that Hck, Lyn and Fgr are viable inhibitor targets for AML therapy, and that selective inhibitors of these kinases may provide therapeutic benefit without the toxicity associated with broad-spectrum kinase inhibitors.

### A-419259 targets multiple AML-associated kinases in vitro and in cells

**Fig 2:**
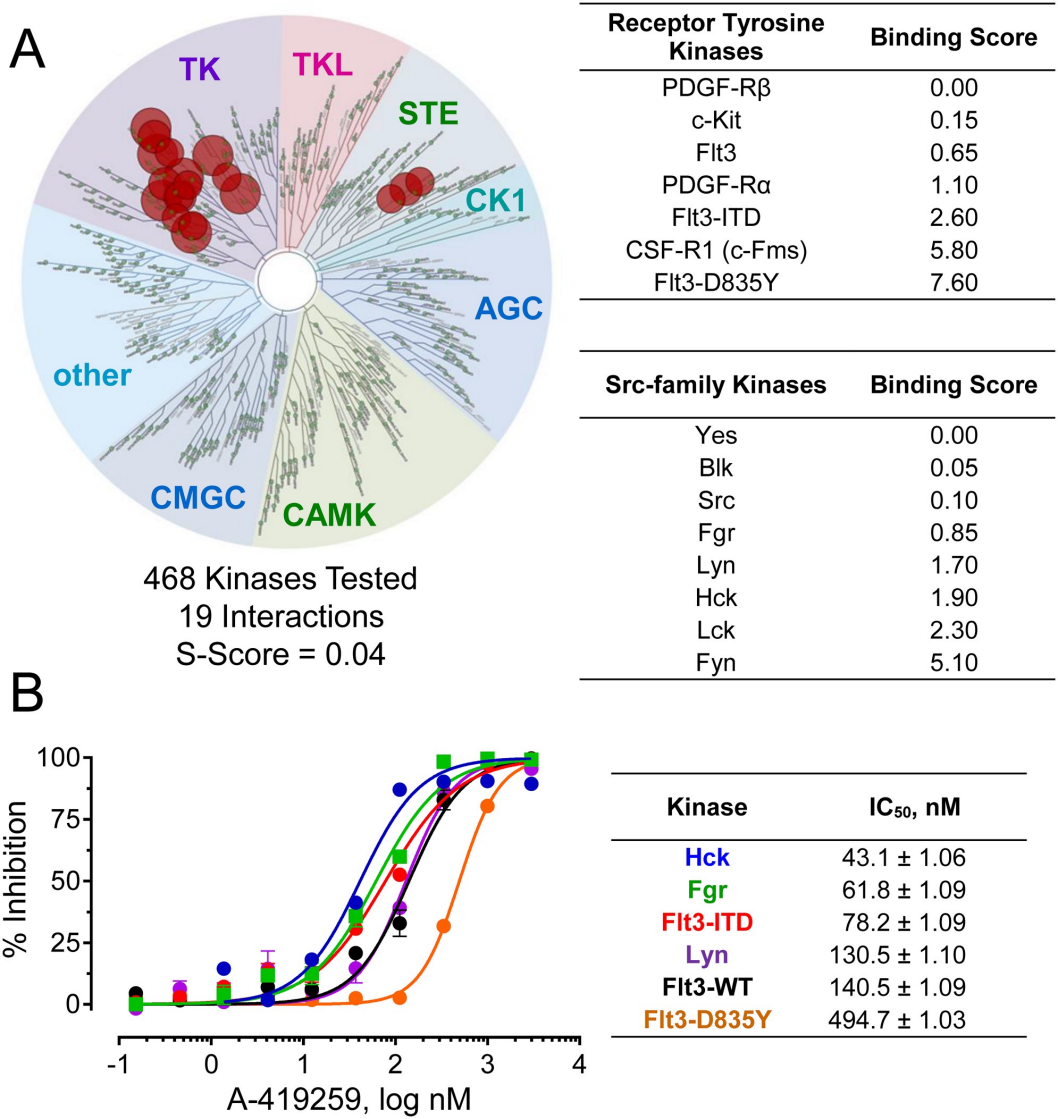
Target kinase specificity profile for the pyrrolopyrimidine tyrosine kinase inhibitor, A-419259. (**A**) KINOMEscan profile of A-419259 tested against 468 kinases at a final concentration of 1 μM. TreeSpot diagram (left) shows all test kinases on a circular dendrogram of the human kinome, with interacting kinases shown as red circles; non-interacting kinases are represented as small green dots. Interacting kinases include class III receptor tyrosine kinases and Src-family kinases, and their individual binding scores are summarized in the tables (right). Each value represents the percent of residual kinase binding to the immobilized probe compound (i.e., a value of 0 represents 100% probe displacement by A-419259). Overall, 19 kinase interactions were observed for an S-Score of 0.04, indicative of a very selective inhibitor. Complete KINOMEscan results are provided in the Supporting Information (S1 Table). (**B**) In vitro kinase assays. Recombinant Flt3 kinase domains (wild type, ITD and D835Y) as well as near-full-length Hck, Lyn and Fgr were assayed using the Z’-LYTE in vitro kinase assay in the presence of a range of A-419259 concentrations, and the resulting data are plotted as percent inhibition relative to the DMSO control (left). The concentration-response curves were best-fit by non-linear regression analysis, and the resulting IC_50_ values are shown in the table as the mean of four replicates ± SE (right).

Previous studies have shown that the pyrrolopyrimidine tyrosine kinase inhibitor A-419259 (also known as RK-20449) has potent anti-tumor efficacy against AML cells both in vitro and in vivo (see Introduction). While initial studies suggested that Hck is the primary inhibitor target for A-419259 in AML [22], additional Src-family kinases as well as Flt3 are also inhibited by this compound in vitro [23]. This raises the important issues of the overall kinase specificity profile for A-419259 and whether inhibition of a single kinase or multiple kinases is responsible for its potent anti-AML effects. To begin to address these questions, we first determined the A-419259 target kinase profile by KINOMEscan, an indirect binding assay that provides kinome-wide assessment of inhibitor specificity [25]. A-419259 was analyzed at the relatively high concentration of 1 μM against 468 kinase targets and showed remarkable overall selectivity with just 19 interactions, indicating that just 4% of the tested kinases bound to the compound. A-419259 interacted most strongly with Src-family kinases, including Hck, Fgr and Lyn, and to several class III receptor tyrosine kinases, including Flt3 (wild-type, ITD and D835Y forms), Kit, the CSF-1 receptor as well as the α and β forms of the PDGF receptor (Fig 2A; complete KINOMEscan results are presented in Supporting Information S1 Table). To validate these results, each of the AML-associated kinases that scored as hits were tested for sensitivity to A-419259 using the Z’Lyte in vitro kinase assay (see Methods). A-419259 inhibited all three AML-associated Src-family members as well as the wild-type and mutant forms of the Flt3 kinase domain (Fig 2B). The IC_50_ values of A-419259 for Hck, Fgr, Lyn and Flt3-ITD varied by three-fold or less, suggesting that inhibition of each of these kinases may contribute to A-419259 anti-AML efficacy.

### Flt3-ITD is a target for A-419259 in AML cells

Our observation that A-419259 potently inhibits Flt3-ITD kinase activity in vitro led us to explore whether Flt3-ITD alone is a target for this compound in AML. To test this hypothesis, we used the human myeloid leukemia cell line TF-1 which is dependent on the cytokine GM-CSF for growth [33]. TF-1 cells do not express endogenous Hck, Fgr or Flt3, and are transformed to GM-CSF-independent growth by the expression of Flt3-ITD but not wild-type Flt3 [34]. In this way, TF-1 cells provided an ideal system for analysis of the contributions of Flt3-ITD, as well as Hck and Fgr, to A-419259 responsiveness.

**Fig 3:**
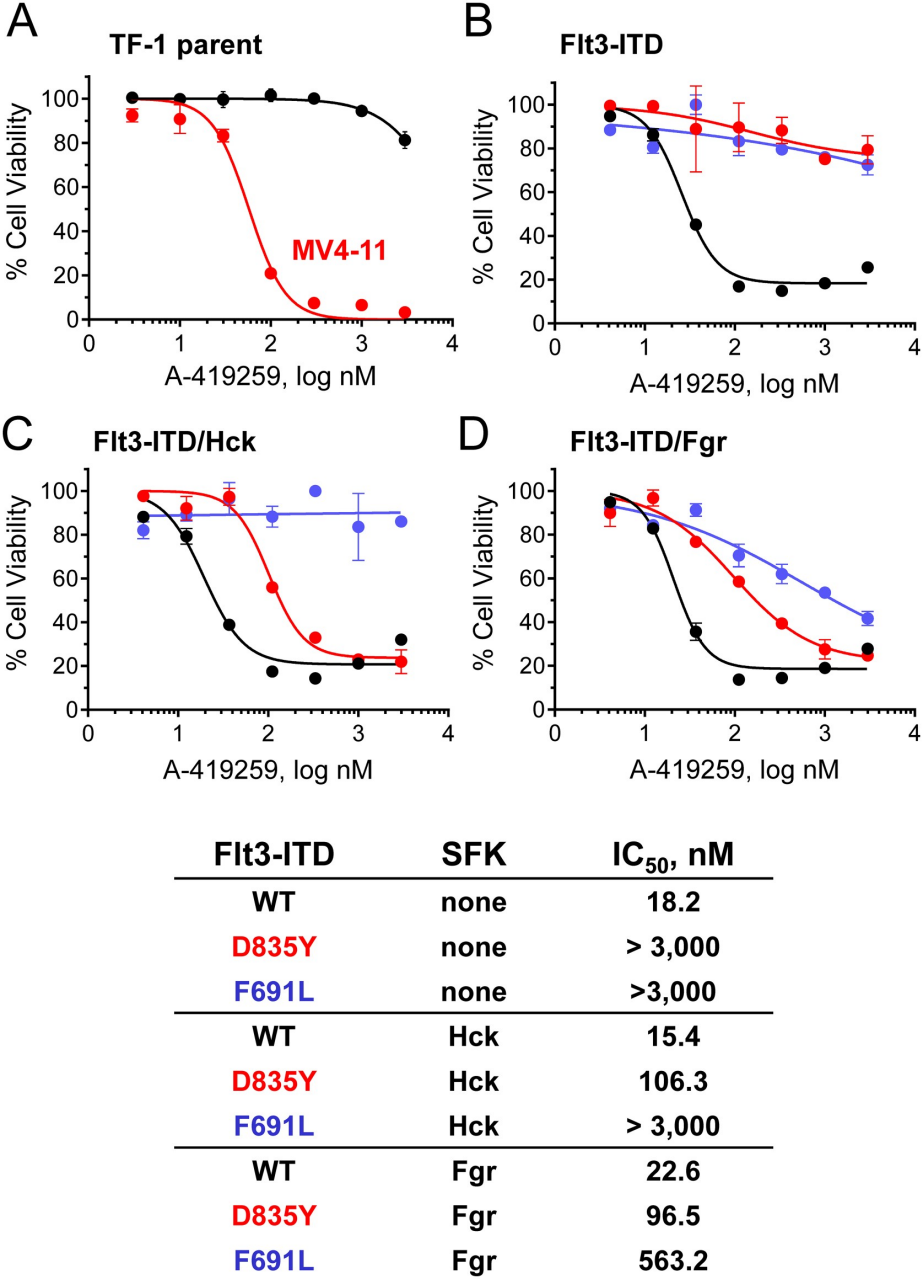
Transformation by Flt3-ITD sensitizes TF-1 myeloid cells to growth suppression by A-419259. TF-1 myeloid cells were transformed to cytokine independence by expression of Flt3-ITD or the inhibitor-resistant mutants D835Y or F691L. These mutations are associated with clinical resistance to quizartinib and other Flt3 kinase inhibitors (see main text). (**A**) The parent TF-1 cell line, as well as the Flt3-ITD^+^ AML cell line MV4-11, were incubated in the presence of a range of A-419259 concentrations or the DMSO carrier solvent (0.1%) alone as control. Cell viability was determined 72 hours later using the CellTiter Blue cell viability assay. Results were normalized to DMSO control values, and are presented as mean percent control ± SD for triplicate determinations. TF-1 cells expressing Flt3-ITD (black curves), Flt3-ITD D835Y (red) or Flt3-ITD F691L (blue) either alone (**B**) or in combination with Hck (**C**) or Fgr (**D**) were assayed for sensitivity to growth arrest by A-419259 as per part A. IC_50_ values for each experiment were determined by non-linear regression analysis and are summarized in the table.

Growth of parental TF-1 cells cultured in the presence of GM-CSF was essentially unaffected by A-419259 treatment, with less than 20% growth inhibition at 3.0 μM (Fig 3A). In contrast, the MV4-11 AML cell line, which is Flt3-ITD^+^ and also expresses active Src-family kinases, was very sensitive to A-419259, yielding an IC_50_ value of 58 nM (Fig 3A). Likewise, TF-1 cells transformed by Flt3-ITD became very sensitive to A-419259 treatment, with an IC_50_ value for growth inhibition of 18.2 nM (Fig 3B). Co-expression of Flt3-ITD with Hck or Fgr did not substantially alter the sensitivity of TF-1 cells to the inhibitor, with IC_50_ values of 15.4 and 22.6 nM, respectively (Fig 3C and 3D). TF-1 cells were then transformed with two established Flt3-ITD inhibitor resistance mutants, D835Y and F691L. The D835Y mutant was chosen because in vitro kinase assays showed about 6-fold resistance to A-419259 (Fig 2), while F691L is the Flt3 gatekeeper residue and mutation of the analogous residue in Hck has been shown to result in A-419259 resistance as described in more detail below. These cell populations were completely resistant to A-419259, supporting direct action of the compound on the Flt3-ITD kinase domain. Interestingly, co-expression of Hck with Flt3-ITD-D835Y (but not F691L) partially re-sensitized the cells to growth arrest by A-419259, with an IC_50_ value just over 100 nM. Co-expression of Fgr re-sensitized both Flt3-ITD-D835Y and Flt3-ITD-F691L cells to A-419259, although the effect on the Flt3-ITD-F691L cells was less pronounced. These results suggest that the presence of Hck and Fgr may suppress the evolution of the Flt3-ITD resistance mutations D835Y and F691L.

**Fig 4:**
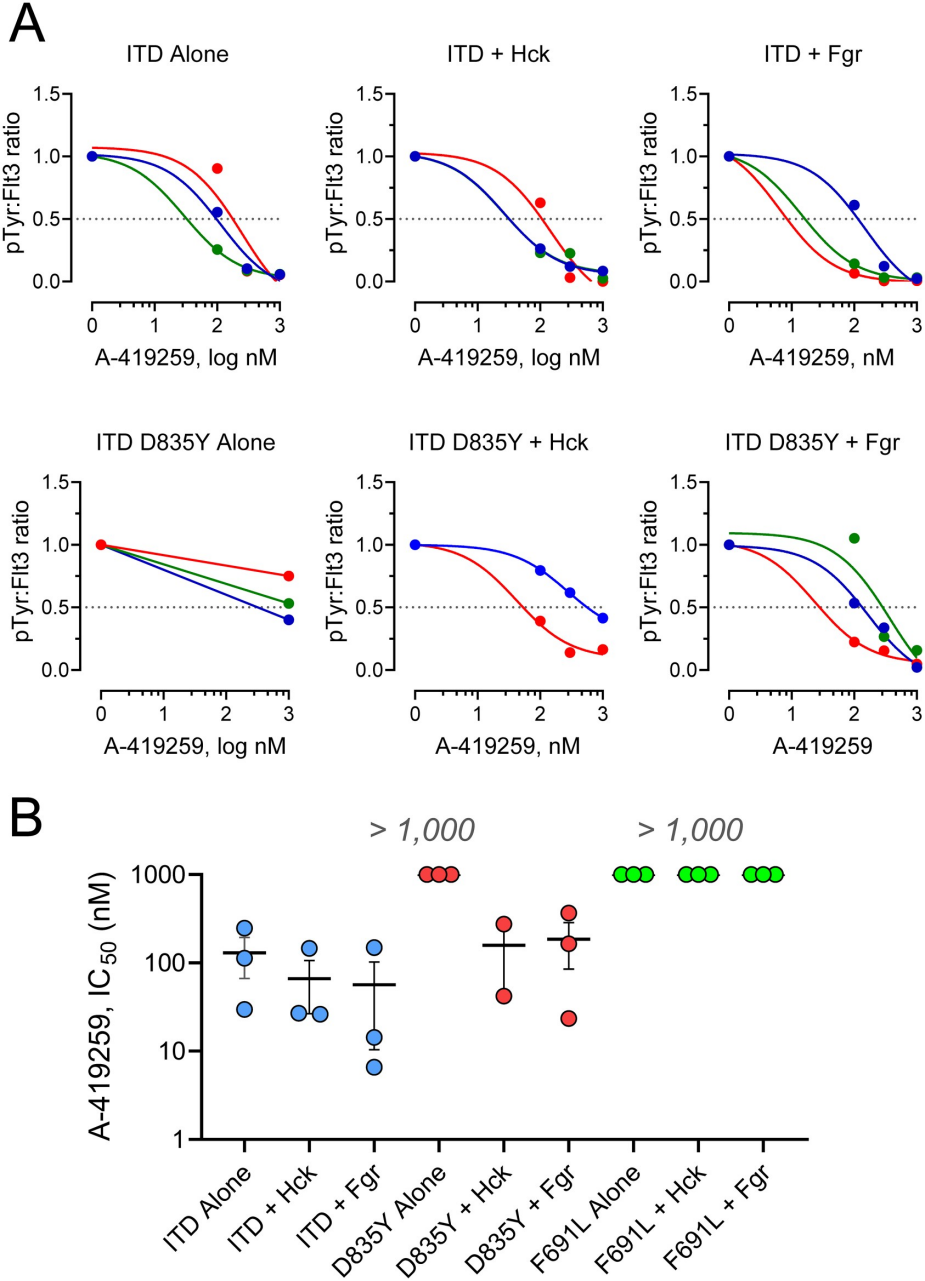
Analysis of Flt3-ITD phosphotyrosine content in TF-1 cells following A-419259 treatment. Each TF-1 cell population from Fig 3 was treated with A-419259 at the concentrations shown or with the DMSO carrier solvent (0.1%) alone as control. Following overnight incubation, Flt3 was immunoprecipitated and analyzed for phosphotyrosine (pTyr) content and Flt3 protein recovery by immunoblotting. Flt3 and pTyr immunoreactivity were quantified using the Odyssey infrared imaging system. Each experiment was performed in triplicate, and the resulting Flt3 pTyr:Flt3 protein ratios were fit by non-linear regression analysis to determine the IC_50_ value. (**A**) Individual concentration-response curves for TF-1 cells expressing Flt3-ITD (ITD), as well as the inhibitor-resistant mutant, D835Y, either alone or in the presence of Hck or Fgr. Each replicate is represented by a different color (red, blue, green). (**B**) Summary of IC_50_ values for all experiments (Flt3-ITD cells, blue; ITD D835Y cells, red; ITD F691L cells, green). Each point represents a single IC_50_ determination, with the vertical bar representing the mean value ± SE. Student’s t-tests did not show a significant difference between the IC_50_ values obtained with TF-1 cells expressing Flt3-ITD alone or in the presence of Hck or Fgr. A-419259 treatment did not inhibit the Flt3 pTyr:Flt3 ratios for cell populations expressing the D835Y mutant alone or the F691L mutant either alone or in the presence of Hck or Fgr (highest concentration tested was 1,000 nM).

To explore the relationship between inhibitor action on cell growth and kinase function, Flt3-ITD was immunoprecipitated from each of the nine cell populations in Fig 3 after A-419259 treatment and immunoblotted for phosphotyrosine content with anti-phosphotyrosine antibodies. Recovery of Flt3-ITD was determined by immunoblotting for Flt3 protein, and phosphotyrosine content was expressed as a ratio of the antiphosphotyrosine to Flt3 protein immunoblot signal intensities following LI-COR imaging of each blot (representative immunoblot images are shown in the Supporting Information, S3 Fig). The phospho-Flt3:Flt3 protein ratios for each of three immunoblot replicates from each population were plotted as individual concentration-response curves, and IC_50_ values were estimated by non-linear regression analysis where possible (Fig 4). Tyrosine phosphorylation of Flt3-ITD was readily observed in TF-1 cells and was inhibited by A-419259 with an average IC_50_ value of 130 nM. Expression of wild-type Hck or Fgr in cells transformed with Flt3-ITD did not significantly change the IC_50_ value for Flt3-ITD phosphotyrosine content (Fig 4B), consistent with the very similar IC_50_ values observed for growth suppression with these three cell populations (shown in Fig 3). However, expression of Hck and Fgr in cells transformed with Flt3-ITD D835Y did show a reduction in the IC_50_ value for Flt3 phosphorylation. Cells expressing the Flt3-ITD D835Y mutant alone yielded an IC_50_ value greater than 1,000 nM, while the Flt3-ITD D835Y cells co-expressing Hck or Fgr showed mean IC_50_ values of 158 and 185 nM, respectively. These observations suggest that the presence of Hck or Fgr re-sensitizes cells transformed with the Flt3-ITD D835Y mutant through a Flt3-dependent mechanism. On the other hand, the presence of Hck or Fgr did not affect Flt3-ITD phosphotyrosine content following A-419259 treatment of cells transformed by the Flt3-ITD F691L mutant (IC_50_ > 1,000 nM in all three cases; Fig 4B). Thus, the partial rescue of growth inhibition in cells co-expressing the F691L mutant with Fgr may be related to transforming signals generated by Fgr itself. Recently, we reported that Fgr is unique among Src-family kinases in that it can induce fibroblast transformation by simple over-expression without mutation, and that it also reduces the requirement of TF-1 cells for GM-CSF when expressed alone [24]. More generally, these studies with TF-1 cells suggest that Flt3-ITD is the primary inhibitor target for A-419259 in Flt3-ITD^+^ AML, although the presence of Hck and Fgr may modulate Flt3-ITD inhibitor sensitivity especially in the presence of Flt3-ITD kinase domain mutations.

### Mutants of Hck and Fgr with engineered resistance reduce AML cell sensitivity to A-419259

**Fig 5:**
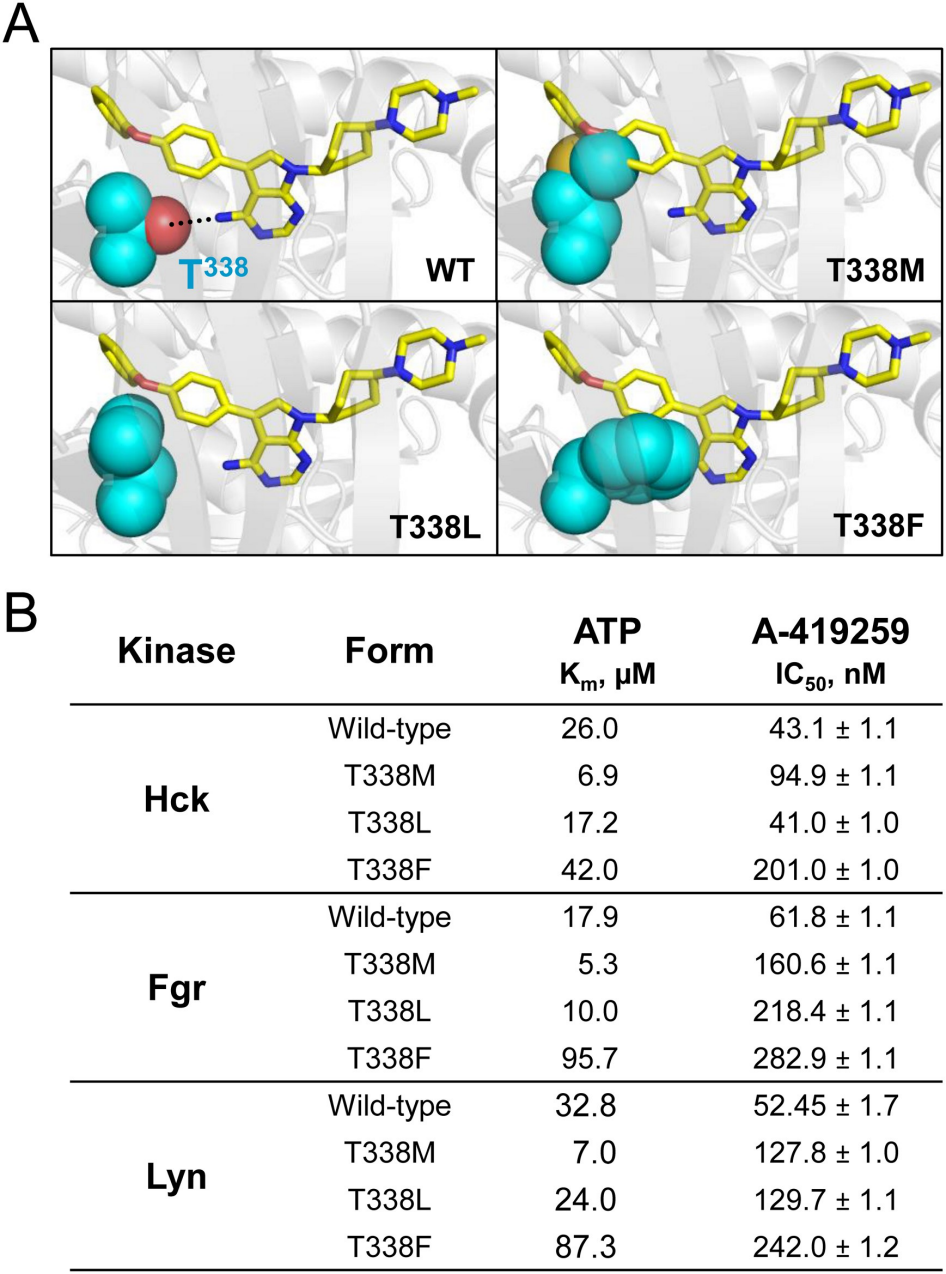
Hck and Fgr gatekeeper mutants confer resistance to A-419259 in vitro. (**A**) Close-up view of the A-419259 binding pocket in the crystal structure of Hck bound to A-419259 (PDB: 4LUE). The carbon backbone of A-419259 is shown in yellow. The side chain of the Hck gatekeeper residue (Thr338; cyan) and forms a hydrogen bond with the primary amine on the pyrrolopyrimidine core of A-419259 (dotted line). Using PyMOL, T338 was substituted with methionine, leucine, and phenylalanine as shown, resulting in loss of the H-bond and steric clash predicted to interfere with inhibitor action. (**B**) Near-full-length Hck, Fgr and Lyn (wild-type and gatekeeper mutants) were expressed in *E*. *coli*, purified and assessed for A-419259 sensitivity in vitro using the Z’-Lyte kinase assay. The K_m_ value for ATP for each kinase was determined first, and subsequent concentration-response experiments with A-419259 were performed with the ATP concentration set to the K_m_ for each kinase. IC_50_ values for A-419259 were determined by non-linear regression analysis of the concentration-response curves, and are shown as the mean ± SE from three independent determinations. Additional kinetic data for all three recombinant kinases is provided in the Supporting Information, S4 Fig.

If Hck, Fgr or other myeloid Src-family members are involved in A-419259 efficacy in Flt3-ITD^+^ AML, then mutations in their drug-binding pockets would be anticipated to confer resistance to A-419259 in AML cells. To test this idea, we engineered A-419259-resistant mutants of Hck and Fgr. The crystal structure of Hck bound to A-419259 (PDB code: 4LUE) reveals that the kinase domain gatekeeper residue, Thr338, forms a key a hydrogen bond with the 4-amino group on the pyrrolopyrimidine heterocycle [35]. Kinase domain gatekeeper residues are well known to confer resistance to many ATP-site kinase inhibitors and can be substituted with alternative amino acids without loss of kinase activity. In the case of Hck, Thr338 is just small enough to allow access of the 4-phenoxyphenyl group of A-419259 to the hydrophobic pocket adjacent to the ATP-binding site. Previous work from our group showed that substitution of T338 in Hck with methionine resulted in resistance to A-419259 both in vitro and in cell-based assays [36]. Here we extended this work by substituting Thr338 in Hck, Fgr and Lyn with larger, more hydrophobic residues, including phenylalanine and leucine in addition to methionine. All of these gatekeeper substitutions are predicted to disrupt hydrogen bonding with the compound and to introduce steric clash, resulting in impaired binding and hence resistance. The position of the gatekeeper residue in the ATP binding site of the Hck kinase domain and the predicted effect of these substitutions are modeled in Fig 5A.

First, we expressed and purified recombinant near-full-length Hck, Fgr and Lyn with each of these gatekeeper mutations, and assessed their kinase activity and inhibitor sensitivity in vitro using the Z’Lyte assay. The wild-type and mutant forms of all 12 recombinant kinases were active, although the gatekeeper mutations altered the K_m_ for ATP in most cases (Supporting Information S4 Fig). With the ATP concentration set to the K_m_ value for each kinase, we then determined the IC_50_ values for A-419259 (Fig 5B). For Hck, substitution of the gatekeeper threonine with methionine or phenylalanine reduced kinase sensitivity to A-419259 by 2-fold and nearly 5-fold, respectively, while leucine substitution was without an effect. For Fgr, all three substitutions resulted in resistance, ranging from 2.5-fold for methionine to nearly 5-fold for phenylalanine. However, none of the Lyn gatekeeper mutants displayed resistance to A-419259, suggesting a different binding mode for the inhibitor with this kinase (Fig 5B). For this reason, Lyn was not explored further in cell-based assays.

We next determined whether expression of wild type or mutant forms of Hck and Fgr affected the growth and survival of TF-1 cells. TF-1 cell populations expressing each kinase were grown in the presence or absence of GM-CSF, and cell viability was assayed daily for five days (Supporting Information S5 Fig). Neither wild type nor any of the gatekeeper mutants of Hck promoted GM-CSF independent growth under these conditions. Expression of wild type Fgr, as well as the T338L gatekeeper mutant, also failed to promote cytokine-independent growth. However, the T338F and T338M mutants of Fgr both transformed TF-1 cells to cytokine independence. The difference between Hck and Fgr in this regard may relate to the intrinsic transforming activity Fgr relative to Hck, which has been linked to differences in its activation loop relative to all other Src-family members [24]. TF-1 cells expressing Flt3-ITD (but not wild type Flt3) grew equally well in the presence or absence of GM-CSF as expected, providing a positive control for this experiment.

**Fig 6:**
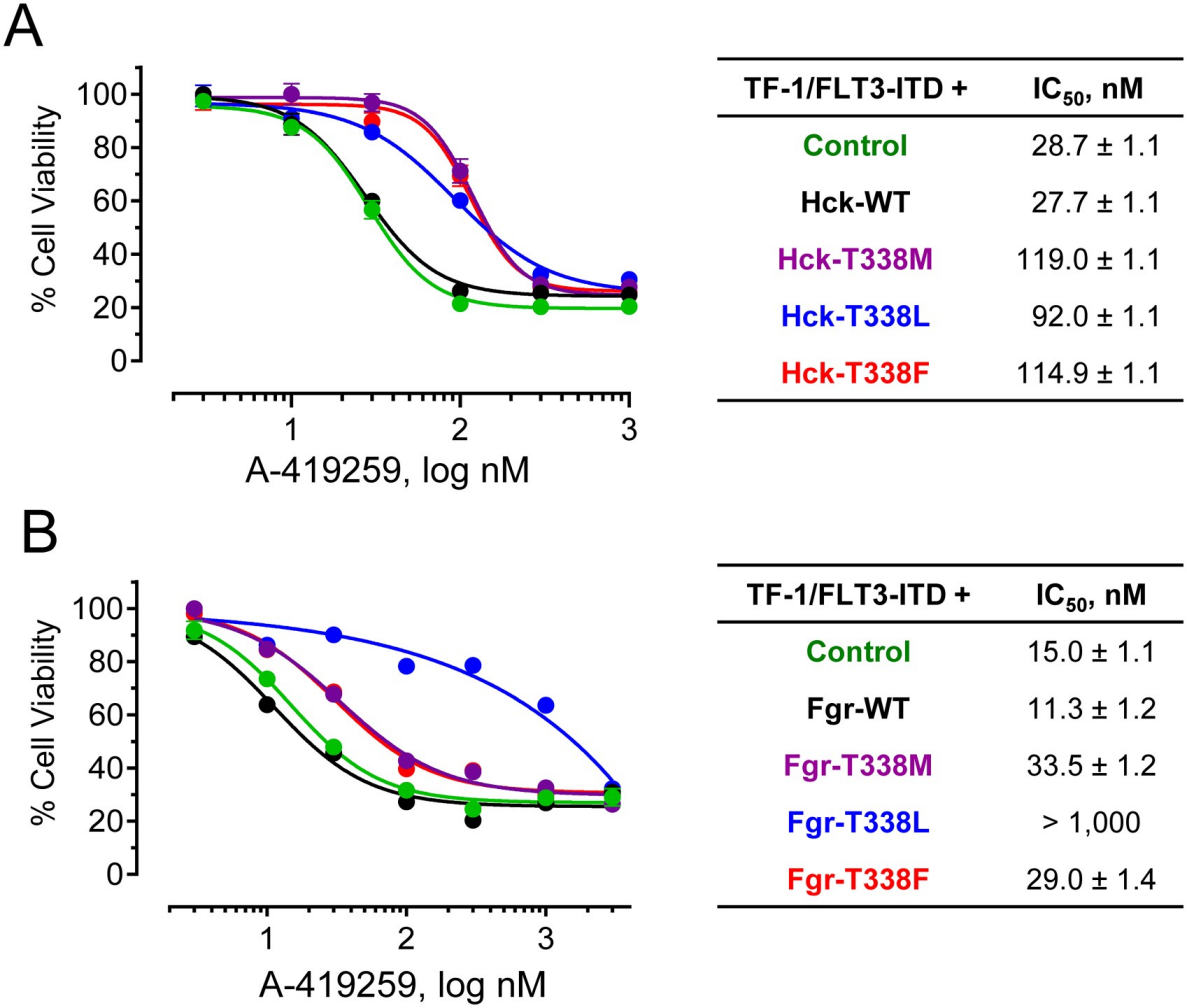
Hck and Fgr gatekeeper mutants confer resistance to A-419259 in TF-1 Flt3-ITD^+^ cells. TF-1 cells co-expressing Flt3-ITD together with wild-type and gatekeeper mutants of Hck (**A**) or Fgr (**B**) were incubated over a range of A-419259 concentrations or the DMSO carrier solvent (0.1%) alone as control. Cell viability was determined 72 hours later using the CellTiter Blue cell viability assay. Results were normalized to DMSO control values, and are presented as mean percent control ± SD for triplicate determinations. IC_50_ values for each experiment were determined by non-linear regression analysis of the resulting concentration-response curves and are summarized in the tables (right).

The wild-type and gatekeeper mutant forms of Hck and Fgr were then stably expressed in TF-1 cells previously transformed by Flt3-ITD. Expression of each Src family kinase did not markedly affect the growth rate of the TF-1/Flt3-ITD cells, with the exception of cells expressing the T338F mutants which grew slightly faster (S6 Fig). With Hck, expression of each of the three gatekeeper mutants resulted in about a 3-fold decrease in cell sensitivity to A-419259 relative to cells expressing the wild-type kinase (Fig 6A). With Fgr, the T338M and T338L mutants also produced about 3-fold resistance to the inhibitor. However, the Fgr-T338L mutant resulted in a much more robust resistance phenotype in the cell-based assay, with an IC_50_ value > 1,000 nM (Fig 6B). Note that expression of wild-type Hck or Fgr had no effect on A-419259 sensitivity compared to TF-1 cells expressing Flt3-ITD alone.

**Fig 7:**
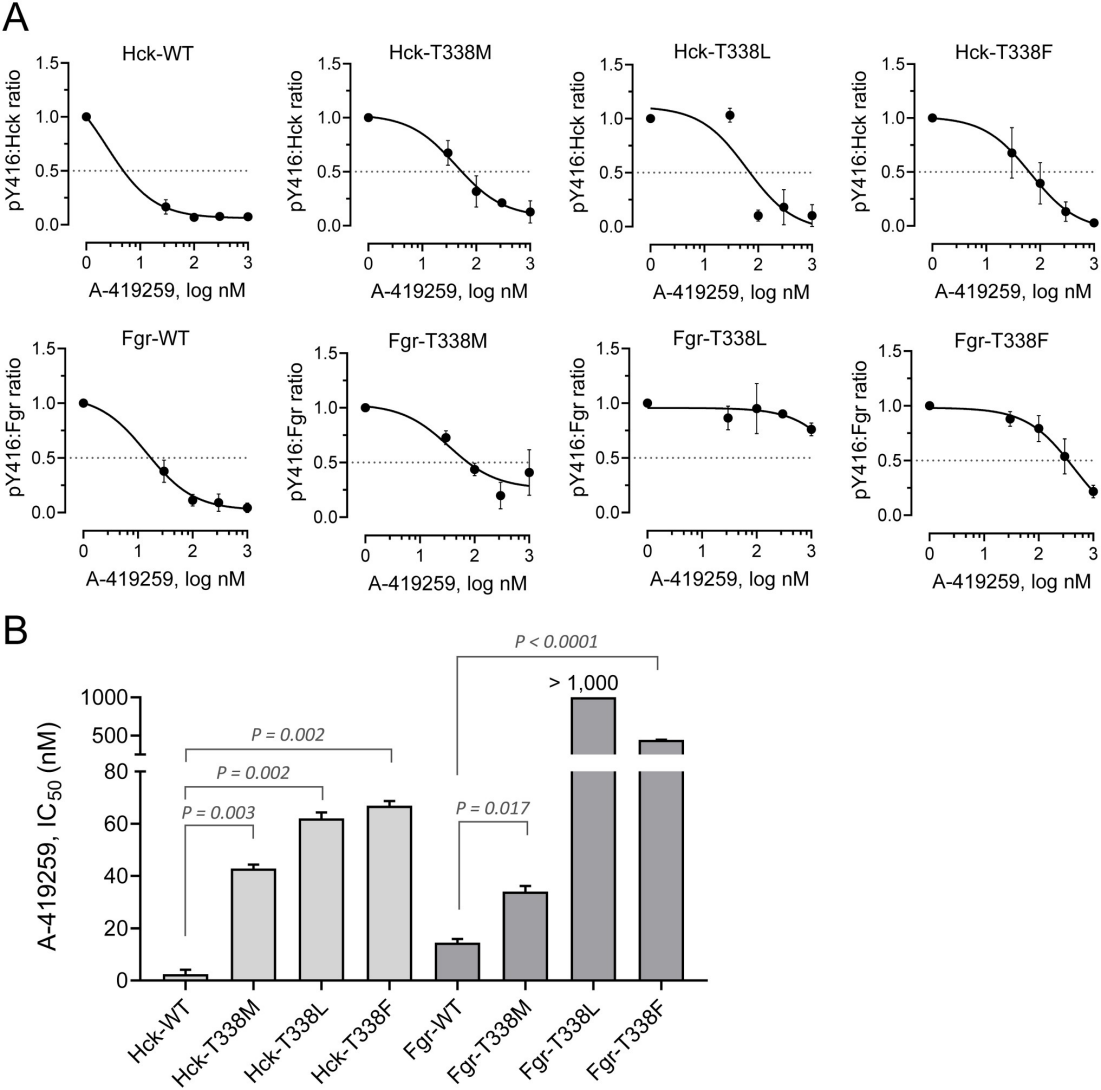
Hck and Fgr gatekeeper mutants remain phosphorylated in the presence of A-419259. TF-1 cells co-expressing Flt3-ITD together with wild type and gatekeeper mutants of Hck or Fgr were incubated overnight with A-419259 at the concentrations shown. Hck and Fgr were immunoprecipitated from clarified cell extracts and immunoblotted for kinase protein recovery as well as activation loop phosphorylation as a marker for kinase activity (pY416). Kinase and pY416 immunoreactivity were quantified using the Odyssey infrared imaging system, and data are plotted as mean pY416:kinase protein ratios ± SE for at least three independent experiments. The resulting concentration responses curves were fit by non-linear regression analysis (**A**) to determine the IC_50_ values ± SE (plotted in **B**). Pair-wise Student’s t tests show significant resistance of each gatekeeper mutant relative to the wild type control; Fgr-T338L could not be compared because the IC_50_ value was greater than the highest concentration tested (1,000 nM).

We next assessed wild-type and mutant Hck and Fgr activity in each of the TF-1 cell populations in the presence of A-419259 by immunoblotting for phosphorylation of Tyr416 (pY416), the site of activation loop phosphorylation found in all Src-family members. Hck and Fgr were immunoprecipitated from each cell population and blotted for both pY416 and kinase protein recovery. The signal intensities of each band were quantified using LI-COR infrared imaging and are expressed as pY416/kinase ratios (Fig 7; representative blot images are shown in the Supporting Information, S7 Fig). TF-1/Flt3-ITD cells co-expressing wild-type Hck or Fgr showed concentration-dependent decreases in Tyr416 phosphorylation in response to A-419259 treatment (Fig 7A), yielding IC_50_ values of 2.4 and 14.5 nM, respectively (Fig 7B). Gatekeeper mutants of Hck expressed in TF-1/Flt3-ITD cells yielded A-419259 IC_50_ values ranging from 40 to 70 nM, consistent with the effect of these mutations on cell growth in the presence of the inhibitor. For the Fgr gatekeeper mutants, pY416 phosphorylation also persisted in the presence of A-419259 treatment, with IC_50_ values significantly higher than the wild-type kinase in each case. This effect was particularly marked for the Fgr T338L mutant, which is consistent with the strong resistance phenotype observed in this cell population. This cell-based observation with Fgr contrasts with the results with recombinant Fgr-T338L in vitro, and may reflect differences in the sensitivity of this mutant to inhibition when expressed as a full-length kinase in the context of the plasma membrane. Taken together, these data with engineered resistance mutants show that inhibition of Hck and Fgr, in addition to Flt3-ITD, is important to the mechanism of action of A-419259.

### Acquired resistance to A-419259 involves mutations to Flt3-ITD but not Src-family kinases in AML cell lines

**Fig 8:**
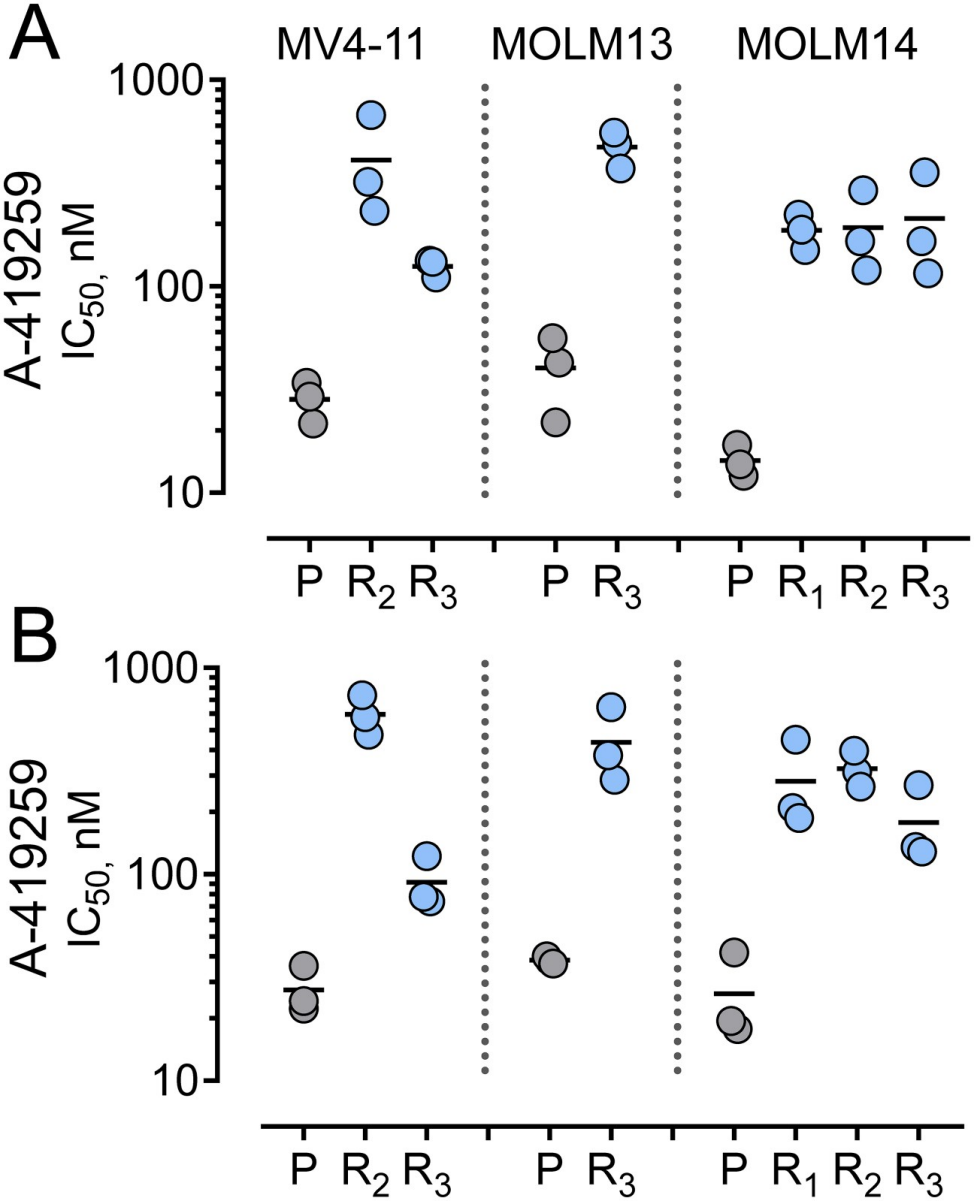
Acquired resistance to A-419259 in the Flt3-ITD^+^ AML cell lines MV4-11, MOLM13 and MOLM14 is a heritable trait. Three independent populations of each AML cell line were passaged in the presence of increasing concentrations of A-419259 until outgrowth was observed with an inhibitor concentration of 1 μM. MV4-11 cells yielded two resistant populations (R_2_, R_3_), MOLM13 yielded one (R_3_), while three were obtained from MOLM14 (R_1_, R_2_, R_3_). P, parent cell line. (**A**) Three replicates of the parent and resistant cell populations were incubated over a range of A-419259 concentrations the DMSO carrier solvent (0.1%) alone as control. Cell viability was determined 72 hours later using the CellTiter Blue cell viability assay. Results are shown relative to the DMSO control values, and IC_50_ values were determined by non-linear regression analysis of the resulting concentration-response curves and are plotted for each cell line. The mean IC_50_ value for each cell population is shown as the black bar, and IC_50_ values for all resistant populations were significantly higher than those for the corresponding parent cell line (p < 0.05 by Student’s t-test). (**B**) Resistant cell populations from part A were passaged 8 times over the course of 4 weeks in the absence of A-419259, followed by re-determination of the IC_50_ values for A-419259. IC_50_ values from parts A and B are summarized in Table 1.

To model how resistance to A-419259 may evolve in vivo, we sequentially passaged three different Flt3-ITD^+^ AML cell lines in the presence of the inhibitor until resistance was observed. We used the human Flt3-ITD^+^ AML cell lines MV4-11, MOLM13 and MOLM14. Importantly, expression of Flt3, Hck, Fgr, and other A-419259 target kinases mirrors that observed in primary bone marrow cells from AML patients (Supporting Information S8 Fig). To generate inhibitor-resistant cell populations, three independent cultures of each cell line were cultured in the presence of A-419259 at a starting concentration of 10 nM. The cultures were incubated until viable cell outgrowth was observed, at which point the concentration of A-419259 was increased by 50%. This process was repeated until outgrowth was observed in the presence of the inhibitor at a concentration of 1 μM, which required 50–80 passages over the course of almost one year. We ultimately obtained two resistant populations from MV4-11, one from MOLM13, and three from MOLM14. IC_50_ values for A-419259 were then determined for each of the resistant cell populations (Fig 8A and Table 1). The selected cells were 5- to 25-fold less sensitive to growth inhibition by A-419259 compared to the parental cell line. To determine whether A-419259 resistance was due to genetic changes, each resistant cell population was passed eight additional times over four weeks in the absence of the inhibitor, followed by re-determination of the IC_50_ values. No significant changes were observed in the degree of inhibitor resistance, consistent with the idea that each population acquired fixed genetic changes that are responsible for resistance as opposed to temporary changes in gene expression, drug efflux or drug metabolism (Fig 8B and Table 1).

**Table 1:** Acquired resistance to A-419259 in Flt3-ITD^+^ AML cell lines.

Cell Population		A-419259 Sensitivity (IC_50_, nM)		Flt3 mutations	
		Initial	Post-holiday	Exome Seq^a^	Sanger (clones)^b^
**MV4-11**	Parent	28.3 ± 3.5	27.5 ± 4.3	none	13/13 WT
	R2	409.8 ± 135.6	596.8 ± 76.3	Asn676 → Ser	6/14 N676S
	R3	125.3 ± 7.3	91.6 ± 15.6	Asn676 → Thr Asp839 → Tyr	8/10 N676T 0/10 D839Y
**MOLM13**	Parent	40.3 ± 9.9	38.4 ± 0.9	None	14/14 WT
	R3	473.1 ± 53.9	436.4 ± 107.3	Asn676 → Ser	3/13 N676S
**MOLM14**	Parent	14.3 ± 1.5	26.4 ± 7.7	None	14/14 WT
	R1	186.7 ± 20.8	282.6 ± 84.2	Asn676 → Ser	5/13 N676S
	R2	192.6 ± 51.4	325.7 ± 38.4	Asn676 → Ser	10/15 N676S
	R3	213.1 ± 73.7	178.3 ± 46.1	Asn676 → Ser	3/11 N676S

Three independent cultures of each parent AML cell line were grown in increasing concentrations of A-419259 until proliferation was observed in the presence of 1 μM compound. Two resistant populations emerged from MV4-11 cells, MOLM-13 produced one, while MOLM-14 produced three. Initial A-419259 IC_50_ values were then determined from concentration-response curves using the CellTiter-Blue assay. The cells were then passaged for 4 weeks in the absence of inhibitor, and IC_50_ values determined again (Post-holiday). IC_50_ values are presented as the mean value ± SE (n = 3). Whole exome sequencing revealed Flt3 mutations in all of the resistant populations, whereas no mutations were observed in Hck, Fgr or other AML-associated tyrosine kinases that are targets for A-419259 as defined by KINOMEscan analysis. Flt3 Asn676 mutations were confirmed in individual clones of Flt3 kinase domain transcripts by Sanger sequencing.

^a^ Missense mutations observed by whole exome sequencing relative to human hg37 reference genome. All nine AML cell populations also exhibited a T227M substitution relative to the reference genome, with the exception of the MV4-11 sensitive and resistant population.

^b^ Partial Flt3 kinase domain sequences were amplified by RT-PCR from total RNA and individual clones were analyzed by Sanger sequencing. Number of mutants/number of clones sequenced are shown.

To explore possible mutations involved in acquired resistance to A-419259, we performed whole exome sequencing of genomic DNA isolated from each parent cell line and the inhibitor-resistant cell populations derived from them. We found that all six A-419259-resistant cell populations acquired missense mutations in Flt3 residue Asn676 while one resistant cell line (MV4-11 R3) also exhibited a Flt3 D839Y mutation, both of which map to the kinase domain (Table 1). To confirm the presence of these Flt3 mutations in each cell population, we amplified Flt3 kinase domain transcripts by RT-PCR from the parent and resistant cells and performed Sanger sequencing on 10–15 individual clones. This analysis confirmed the presence of Flt3 Asn676 mutations in all six resistant populations, but not in any Flt3 clones from the parent cells. In contrast, the Flt3 D839Y mutation observed by whole exome sequencing of MV4-11 R3 cells was not present in any of ten independent clones.

Whole exome sequencing revealed an average of 25,050 mutations in each resistant cell population relative to the corresponding parent cell line. Of these, an average of 15,738 mutations localized to protein coding sequences, although only a few non-synonymous mutations were observed in other A-419259 target kinases identified in the KINOMEscan profile. MOLM14 R1 had an S1060C mutation in the receptor tyrosine kinase, ErbB3. This mutation is C-terminal to the kinase domain of ErbB3 and is therefore unlikely to be involved in inhibitor resistance. MV4-11 R3 had an R428N mutation in Mknk2, which is also C-terminal to the kinase domain. Finally, MV4-11 R2 had a C280S mutation in Src, which localizes to the non-catalytic SH2 domain. Our qPCR analysis shows that Src is expressed at very low levels in parental MV4-11 cells and even lower levels in MV4-11 R2 cells (Supporting Information S8 Fig). While a mutation in the Src SH2 domain could potentially influence A-419259 sensitivity through an allosteric mechanism, the low overall Src expression argues against a role for Src in A-419259 action and resistance in these cells. No mutations were observed in any other A-419259 target kinases identified by KINOMEscan and expressed in these AML cell lines, including Hck, Lyn and Fgr.

**Fig 9:**
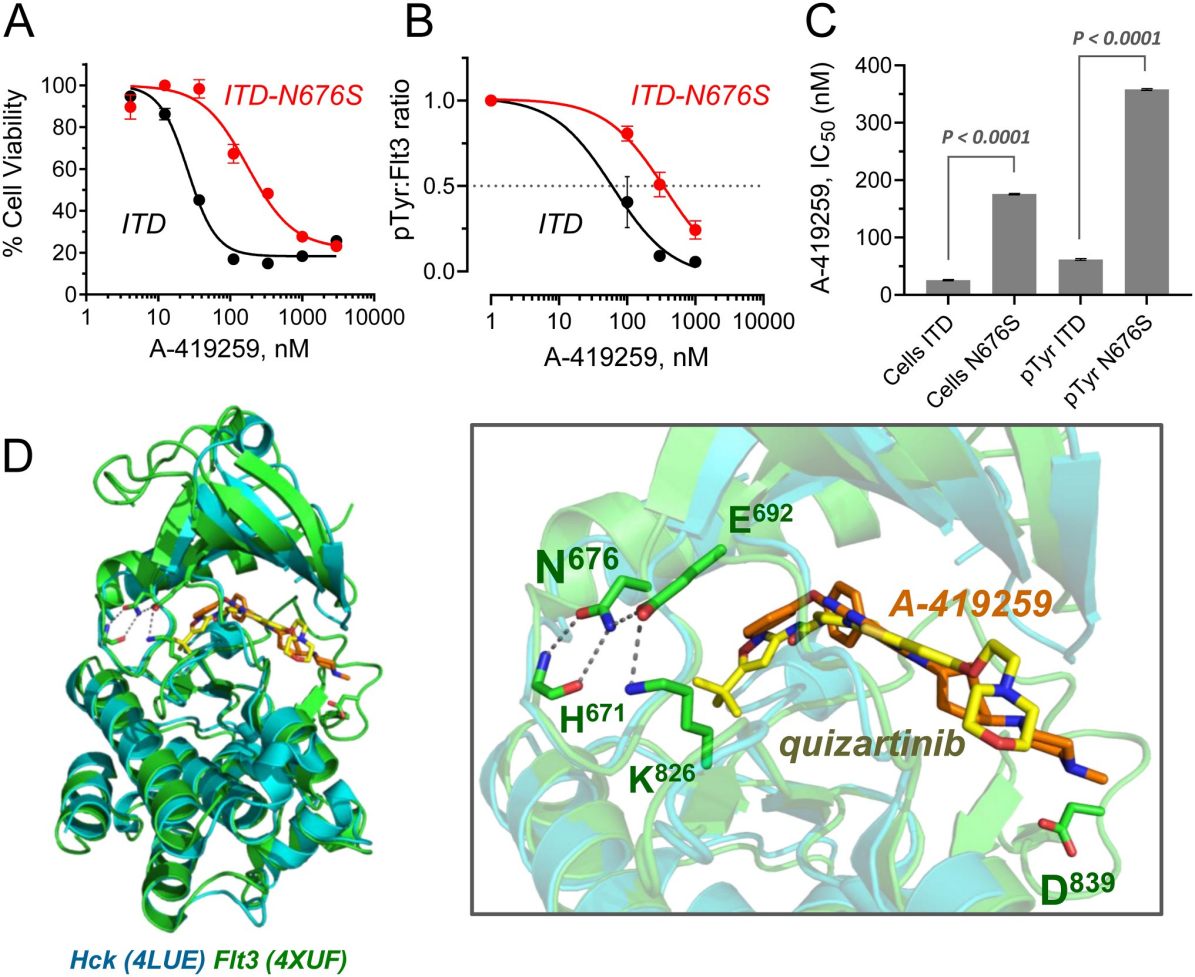
Flt3-ITD N676S mutation confers resistance to A-419259 in TF-1 cells. (**A**) TF-1 cells expressing Flt3-ITD or the Flt3-ITD N676S mutant were incubated over a range of A-419259 concentrations or the DMSO carrier solvent (0.1%) alone as control. Cell viability was determined 72 hours later using the CellTiter Blue cell viability assay. Results are shown relative to the DMSO control values, and IC_50_ values were determined by non-linear regression analysis of the resulting concentration-response curves. (**B**) Cell populations from part A were incubated overnight in the presence of the A-419259 concentrations shown. Flt3 was then immunoprecipitated and analyzed for phosphotyrosine (pTyr) content and Flt3 protein recovery by immunoblotting followed by Odyssey infrared imaging. The mean pTyr:Flt3 protein ratios ± SE for three independent experiments were fit by non-linear regression analysis. (**C**) The IC_50_ values for growth inhibition (Cells) and Flt3 phosphotyrosine context (pTyr) are shown, and were compared for statistical significance by Student’s t test. (**D**) Model of A-419259 bound to the Flt3 kinase domain. The crystal structure of the Flt3 kinase domain bound to quizartinib (PDB: 4XUF; green) was aligned with the crystal structure of the Hck kinase domain bound to A-419259 (PDB: 4LUE; blue) using PyMol. The overall alignment is shown at left, and a close-up of the inhibitor binding site is shown at right. Significant overlap was observed in the positions of quizartinib (yellow) and A-419259 (orange) in the ATP-binding site. In the Flt3 structure, residue Asn676 forms a local network of polar contacts with the side chains of Glu692, Lys826 and the main chain of His671. Acquired mutations in this residue were associated with resistance to A-419259 in AML cell lines. Whole exome sequencing also identified a substitution of Flt3-ITD Asp839 (shown) in MV4-11 population R_3_; this mutation was not verified in subsequent Sanger sequencing of individual clones (Table 1).

To determine whether the Flt3 N676S point mutation was sufficient to confer resistance to A-419259, Flt3-ITD N676S was expressed in TF-1 cells. Each cell population was then compared to control TF-1/Flt3-ITD cells for sensitivity to growth arrest by A-419259. Cells expressing the Flt3-ITD N676S mutant were significantly less sensitive to A-419259 than cells expressing Flt3-ITD, with IC_50_ values of 176 and 26 nM, respectively (Fig 9A). The mutant kinase also showed significantly reduced sensitivity to A-419259 in terms of phosphotyrosine content, consistent with the resistant phenotype (Fig 9B and 9C). Asn676 is located near the active site in a crystal structure of the Flt3 kinase domain (Fig 9D) and is positioned to impact inhibitor binding through an allosteric mechanism (see Discussion). We also evaluated the impact of Hck and Fgr expression on TF-1 cells expressing the Flt3-ITD N676S resistance mutation for A-419259. Neither Hck nor Fgr co-expression markedly affected the inhibitor sensitivity of TF-1 cells expressing the Flt3-ITD N676S mutant (Supporting Information, S9 Fig).

### Evaluation of A-419259 target kinase gene expression in resistant AML cells

While mutation of Flt3-ITD N676 was consistently observed across six independent A-419259-resistant AML cell populations, the possibility exists that changes in the expression of Hck, Fgr, or other target kinases for this inhibitor may also contribute to the resistant phenotype. To address this possibility, we used quantitative real-time RT-PCR to determine the relative expression profiles of all A-419259 target kinases identified by KINOMEscan analysis in each parent and resistant cell population. We also profiled several additional kinases previously identified as targets for TL02-59, another AML-active kinase inhibitor with a slightly broader target specificity profile than A-419259 [20]. Of the 27 kinases profiled, only the non-receptor tyrosine kinase Syk was consistently upregulated in the A-419259-resistant cell populations (Supporting Information, S10 Fig). Previous studies have implicated Syk in the pathogenesis of AML, and upregulation of Syk is known to confer resistance to other Flt3 inhibitors [37]. To determine whether the upregulation of Syk kinase activity contributes to A-419259 resistance, we used the Syk inhibitor PRT 062607 [38]. All three parent AML cell lines, as well as their resistant counterparts, were sensitive to growth suppression by this Syk inhibitor, with IC_50_ values in the 0.5 to 2.0 μM range (Supporting Information, S11 Fig). We then performed concentration-response studies with A-419259 in the presence of fixed concentrations of the Syk inhibitor (50, 100 and 200 μM) using the CellTiter-Blue cell viability assay. Overall, addition of PRT 062607 did not affect sensitivity to A-419259, suggesting that upregulation of Syk expression does not contribute to A-419259 resistance. One exception was the R2 population of A-419259-resistant MV4-11 cells, where a subtle but significant leftward shift in the A-419259 concentration-response curve was observed with increasing concentrations of the Syk inhibitor.

## Discussion

Flt3 is a *bona fide* proto-oncogene in the context of AML, which has led to the development of multiple Flt3 kinase inhibitors for evaluation as targeted therapy [7,14]. The myeloid Src-family kinases Hck, Lyn and Fgr have been independently described as relevant AML drug targets, and inhibition of these kinases is also a promising strategy for AML treatment [19,20,22,34]. In the present study, we demonstrate that these myeloid Src-family members are highly expressed in a substantial subset of AML patients (Fig 1A). Furthermore, Hck and Fgr expression are highly correlated (Supporting Information S1 Fig), suggesting that the subset of AML patients dependent on Src-family kinase signaling will be most susceptible to selective inhibitors of these kinases. Along these lines, Hck and Fgr as well as Lyn mRNA expression are strong predictors of AML patient prognosis, while Flt3 expression *per se* is not (Fig 1B). These observations led us to investigate the efficacy of Src-family kinase inhibition in the context of Flt3-ITD^+^ AML. We focused primarily on Hck and Fgr since their expression is limited to myeloid cells, while Lyn is more ubiquitously expressed.

Hck expression is upregulated in chemotherapy-resistant AML leukemic stem cells [21], and inhibition of Hck with the ATP-competitive kinase inhibitor A-419259 prevented the engraftment of primary AML cells in genetically immunocompromised mice [22]. Here we show that that A-419259 inhibits not only Hck, but also Fgr and other Src-family kinases in addition to Flt3 (Fig 2). This observation raised the question of whether or not A-419259 efficacy could be attributed solely to Hck inhibition. To explore this question, we developed a model system based on the human myeloid cell line, TF-1. These cells do not express detectable Hck or Fgr but are readily transformed to cytokine-independent growth following retroviral transduction with Flt3-ITD. We found that TF-1 cells transformed with Flt3-ITD became very sensitive to A-419259 treatment, while those expressing two common Flt3 inhibitor resistance mutants (D835Y and F691L) were completely insensitive to this compound (Fig 3). Interestingly, co-expression of Hck partially re-sensitized TF-1/Flt3-ITD-D835Y cells to A-419259, while co-expression of Fgr re-sensitized cells expressing either of these inhibitor-resistant Flt3-ITD mutants. These data demonstrate that the anti-AML efficacy of A-419259 is dependent on inhibition of Flt3-ITD and myeloid Src-family kinases when they are co-expressed in the same AML cell population. D835Y and F691L are among the most frequent Flt3-ITD clinical resistance mutations for highly selective Flt3 inhibitors such as quizartinib, which does not inhibit myeloid Src-family kinases (S. Hellwig and T. Smithgall, unpublished data). Our findings with A-419259 suggest that combined inhibition of Flt3-ITD and Src-family kinases may delay the appearance of resistance in the clinic by simultaneously inhibiting the activity of both kinase families.

As a second approach to validate Hck, Fgr and Lyn as relevant targets for A-419259 in AML, we developed kinase mutants with engineered resistance to this inhibitor (Fig 5). Using the crystal structure of Hck bound to A-419259 (PDB 4LUE) as a guide [35], we substituted the gatekeeper residue in each kinase domain (Thr338) with methionine, leucine or phenylalanine. Each mutation conferred resistance to recombinant kinase mutants of Hck and Fgr in vitro, most likely due to loss of a hydrogen bond with the inhibitor and increased steric bulk at the binding site. Unexpectedly, gatekeeper mutants of Lyn retained A-419259 sensitivity, suggesting a distinct binding mode for A-419259 in this case. Expression of the inhibitor-resistant Hck and Fgr mutants in TF-1 cells transformed with Flt3-ITD decreased their sensitivity to A-419259 (Figs 6 and 7), providing direct evidence that these Src-family members are important for inhibitor action. These results are reminiscent of earlier studies of this compound in the context of CML cell lines. In this case, a gatekeeper mutant of Hck (T338M) also resulted in A-419259 resistance, establishing a role for this kinase in Bcr-Abl signaling as well [36].

To investigate possible pathways to A-419259 resistance in an unbiased manner, we generated de novo resistance to A-419259 using the Flt3-ITD^+^ AML cell lines MV4-11, MOLM13 and MOLM14. Each cell line was cultured with increasing concentrations of A-419259 over many months, resulting in six independent cell populations able to grow in the presence of 1 μM A-419259, which is 25–70 times the IC_50_ value for growth suppression of the parent cell lines. Repeated passage of each resistant population in the absence of A-419259 did not result in loss of resistance, suggesting that acquired heritable mutations are responsible for the resistant phenotype (Table 1).

To explore the genetic basis of resistance, whole exome sequence analysis was performed on genomic DNA isolated from each parent cell line and their inhibitor-resistant progeny. All six resistant cell lines exhibited missense mutations of Flt3 Asn676, while Hck, Fgr and almost all other A-419259 target kinases identified by KINOMEscan analysis were wild-type. The Flt3 Asn676 mutations were validated by Sanger sequencing of individual Flt3 clones isolated by RT-PCR from each resistant population; no mutations were observed in the parent cell lines. Transformation of TF-1 cells with Flt3-ITD bearing the N676S mutant showed significant resistance to A-419259, validating this mutation as a likely mechanism of acquired resistance to A-419259.

Experiments with TF-1 cells transformed by Flt3-ITD bearing the clinical inhibitor resistance mutations D835Y and F691L were completely resistant to A-419259 (Fig 3). However, we were unable to detect either of these mutations in AML cell populations with acquired resistance to A-419259. One possible explanation may relate to our observation that TF-1 cells expressing Flt3-ITD D835Y or F691L are re-sensitized to A-419259 by co-expression of Hck or Fgr, while cells expressing the F691L mutant were partially rescued by Fgr. Unlike TF-1 cells, MV4-11, MOLM13 and MOLM14 cells all express endogenous Hck and Fgr, which may suppress the evolution of resistance via Flt3-ITD D835Y and F691L mutations. In contrast, co-expression of Hck or Fgr had little impact on the A-419259 resistance of TF-1 cells transformed by Flt3-ITD N676S, consistent with this idea.

The Flt3 Asn676 mutation has been linked to clinical resistance to quizartinib, midostaurin, and other Flt3 inhibitors [15,39]. In the crystal structure of quizartinib bound to the Flt3 kinase domain (PDB: 4XUF), Asn676 forms a hydrogen bond network with three adjacent residues near the inhibitor binding site: His671, Glu692, and Lys826 [15]. This structure led to the hypothesis that mutation of Asn676 disrupts this hydrogen bond network to favor the active, ‘DFG-in’ state of the active site, thus resulting in resistance to quizartinib and other so-called ‘Type II’ inhibitors with conformationally sensitive binding modes. Alignment of the kinase domains from the crystal structure of Hck bound to A-419259 (PDB: 4LUE) with quizartinib-bound Flt3 shows remarkable overlap in the position of the two inhibitors (modeled in Fig 9C). This alignment suggests that Asn676 mutations may affect A-419259 binding to the Flt3 kinase domain in a manner similar to that as quizartinib.

Recent work from Yuan *et al*. describes synthesis and optimization of another Flt3-ITD kinase inhibitor series based on a pyrrolopyrimidine core with clinical promise for AML [40]. Interestingly, KINOMEscan data show that the most active analog (compound 9u) interacts with both Hck and Fgr in addition to Flt3, suggesting that it may possess activity against multiple AML-associated kinases in the same way as A-419259. While both 9u and A-419259 compounds share a pyrrolopyrimidine scaffold, they are structurally quite distinct, with compound 9u bearing a much closer relationship to quizartinib than A-419259. That said, compound 9u, unlike A-419259, is a very potent inhibitor of quizartinib-resistant forms of Flt3 (D835 and F691 mutants). However, 9u was not tested against the Flt3 N676S mutant, which was the only resistance mutation that we observed following long-term acquired resistance studies with A-419259 in three independent AML cell lines. It will be very interesting to see what mutations arise in Flt3-ITD^+^ AML cells following evolved resistance to this new inhibitor.

In summary, our study provides new evidence that the level of Hck, Lyn and Fgr expression has strong prognostic power in AML. We also demonstrate that the anti-leukemic efficacy of the tyrosine kinase inhibitor A-419259 is dependent on inhibition of myeloid Src-family kinases as well as Flt3 in the context of Flt3-ITD^+^ AML. Importantly, the ability of A-419259 to inhibit both Flt3-ITD and myeloid Src family kinases may suppress the evolution of common resistance mutations seen for other Flt3 inhibitors, especially D835Y, although the N676S mutation is still a liability with A-419259. Combination therapy with Flt3 inhibitors that display non-overlapping acquired resistance profiles may suppress a broader range of resistance mutations. For example, quizartinib and A-419259 have minimally overlapping resistance profiles, since quizartinib resistance primarily involves D835Y and F691L mutations while A-419259 resistance primarily involves substitution of Asn676 as established here.

## Supporting information

S1 Fig Pairwise correlation analysis of Hck, Fgr and Lyn transcript levels across AML samples in the TCGA cohort. (**A**) Hck, Fgr, and Lyn transcript levels are shown as the number of kinase cDNA fragments per kilobase of transcript per million mapped reads for all AML patients in the TCGA cohort (n = 163). Dots represent individual patient expression data, with the dot color representing Flt3 mutational status (grey, wild type; red, ITD; blue, D835Y). The plots compare Fgr vs. Hck (*left*), Lyn vs. Hck (*middle*) and Lyn vs. Fgr (*right*). Shown below each plot is the Pearson correlation coefficient (r) and p-value for each comparison. Code used to generate these plots is available on GitHub as described under Materials and Methods. (**B**) Distribution of Hck, Fgr, and Lyn expression levels in the presence of common AML mutations, including FLT3, NPM1, IDH1/2, DNMT3A, RUNX1, p53, NRAS, CEBPA, WT1, and PTPN11. Kinase transcript levels are shown as box and whisker plots, where the box defines the 25-75^th^ percentile, the vertical bar is the median value, and the whiskers show the 5^th^ to 95^th^ percentiles. Each gene symbol includes all mutations present within that locus, with the exception of Flt3-ITD and Flt3-D835Y which are specific mutations. RUNX1T1 is the RUNX1-RUNX1T1 translocation associated with AML.
(PDF)

S2 Fig Comparison of Hck, Fgr and Lyn transcript levels across all tumors in the TCGA cohort. Transcript levels for Hck, Fgr and Lyn were downloaded for all tumors available on cBioPortal from the TCGA database. Data are shown as the number of cDNA fragments per kilobase of transcript per million mapped reads (FKPM). Each box and whisker plot shows the median value (middle line in box), 25^th^-75^th^ percentiles (edges of box), and outliers (whiskers) for each data set. Code used to generate these plots is available on GitHub as described under Materials and Methods.
(PDF)

S3 Fig Representative anti-phosphotyrosine immunoblots from TF-1 cells expressing Flt3-ITD alone or with Hck and Fgr. Each of the TF-1/Flt3-ITD cell populations indicated at the top (wild-type; D835Y; F691L ± wild-type Hck or Fgr) were treated with A-419259 at the nM concentrations shown or with 0.1% DMSO (carrier solvent) as control. Following overnight incubation, Flt3 was immunoprecipitated and analyzed for phosphotyrosine content by immunoblotting. Anti-phosphotyrosine immunoreactivity was detected using the Odyssey infrared imaging system. Positions of molecular weight markers (M) are shown in kDa. The phosphorylated Flt3-ITD bands are indicated by the arrows; in some cases, a lower molecular weight Flt3 band is observed which corresponds to the unglycosylated form of the receptor. Control blots were performed with anti-Flt3 antibodies to verify kinase recovery (lower panel in each set); the major Flt3 species recovered is the unglycosylated form (*arrows*). Band intensities for phosphorylated Flt3 were normalized to the Flt3 protein levels from at least three 3 independent experiments, and the ratios were used to generate the IC_50_ values shown in Fig 4.
(PDF)

S4 Fig *In vitro* kinetic analysis of wild-type and gatekeeper mutants of Hck, Fgr and Lyn. Recombinant near-full-length kinases, consisting of the SH2, SH3 and kinase domains plus the negative regulatory tail, were expressed in *E*. *coli* in the presence of Csk (to phosphorylate the tail tyrosine) and PTP1B (to keep the activation loop dephosphorylated). Purified kinases were assayed *in vitro* using the Z’-LYTE kinase assay (ThermoFisher) and the Tyr-2 peptide substrate (final concentration of 1.0 μM). **A**) Determination of K_m_ values for ATP. Kinase activity was determined over the range of ATP concentrations shown. Reaction velocities were determined by quenching each reaction at various time points. The resulting curves were fit to the Michaelis-Menten equation using GraphPad Prism v7.04, and the resulting K_m_ values are shown in the Table at right. **B**) Determination of intrinsic kinase activity. Each kinase was assayed over a range of input amounts with the ATP concentrations set to the K_m_. Kinase titration curves were best-fit by non-linear regression analysis (Prism) and the resulting EC_50_ values are shown in in the table. Kinase forms color-coded as per the Table are also used in the plots in part A and B.
(PDF)

S5 Fig Fgr but not Hck gatekeeper mutants transform TF-1 myeloid cells to cytokine-independent growth. Wild-type and gatekeeper mutants of Fgr and Hck were stably expressed in TF-1 cells. After selection with G418, cells were cultured in the presence or absence of GM-CSF and viability was monitored daily using the CellTiter Blue assay (Promega). Data are presented as relative fluorescence units, which increase as a function of cell proliferation. TF-1 cells transformed with Flt3-ITD served as a positive control, while cells transduced with an empty vector served as negative control. Expression of each kinase was confirmed by immunoblotting (*data not shown*). TF-1 cells expressing Fgr-T338M showed GM-CSF-independent proliferation to the same extent as Flt3-ITD, while Fgr-T338F produced a partial cytokine-independent phenotype.
(PDF)

S6 Fig Expression of Fgr and Hck gatekeeper mutants in TF-1/Flt3-ITD cells does not markedly affect their growth. Wild-type and Thr338 gatekeeper mutants of Hck and Fgr were expressed in TF-1/Flt3-ITD cells using recombinant retroviruses with a second selection marker (see Materials and methods). Each cell population was then plated at equal density, and cell viability was assessed 48 h later using the Cell Titer Blue assay. Raw fluorescence values were normalized to those obtained with control TF-1/Flt3-ITD cells. Each population was assayed in triplicate, and the average values are shown ± SEM. End-point proliferation varied by less than 15% relative to the control TF-1/Flt3-ITD cells, with the exception of the cells expressing the Hck and Fgr T338F mutants, which showed increases of 24% and 54%, respectively (p < 0.0001 by Student’s t-test in each case).
(PDF)

S7 Fig Representative immunoblots of activation loop tyrosine phosphorylation of Hck and Fgr gatekeeper mutants in A-419259-treated TF-1 cells. TF-1 cells co-expressing Flt3-ITD together with wild-type and gatekeeper mutants of Hck (*upper panels*) or Fgr (*lower panels*) were incubated overnight with A-419259 at the nM concentrations shown. Hck and Fgr were immunoprecipitated from clarified cell extracts and immunoblotted for activation loop phosphorylation as a marker for kinase activity (pY416; upper panels in each set) as well as kinase protein recovery (lower panels in each set). Kinase protein and pY416 immunoreactivity were quantified directly using the Odyssey infrared imaging system. Representative uncropped pY416 and kinase protein immunoblot images are shown, along with molecular weight markers in kDa (*M*). For the pY416 blots, the positions of the pY416 bands are indicated by the arrows (pHck and pFgr); in most cases, the heavy chains of the anti-Hck and anti-Fgr antibodies used to immunoprecipitate the kinases are also observed (*Ig*_*H*_; *grey arrows*). Replicate blots were used to generate the IC_50_ values shown in Fig 7.
(PDF)

S8 Fig The Flt3-ITD^+^ AML cell lines MV4-11, MOLM13 and MOLM14 express similar levels of A-419259 target kinases as AML patient cells. Relative expression of A-419259 target kinases (based on KINOMEscan profiling) was determined in a cohort of 26 AML patient bone marrow samples using quantitative real-time RT-PCR (qPCR). Relative expression values are represented as box-and-whisker plots, with statistical outliers (value > 3σ) represented as grey diamonds. A description of these patient samples and the methods used to determine the relative kinase expression profiles is reported in detail in Weir, *et al*. *ACS Chem*. *Biol*. 23:1551, 2018; PMID: 29763550. Kinase expression profiles in the three AML cell lines were determined using the same qPCR approach, and these values are overlaid on the patient data using the color-coded dots as indicated. Code used to generate these plots is available on GitHub as described under Materials and Methods.
(PDF)

S9 Fig Expression of Hck or Fgr does not markedly influence sensitivity of TF-1/Flt3-ITD N676S cells to A-419259. TF-1 cells were transformed to GM-CSF independence by expression of the N676S mutant of Flt3-ITD. Wild-type Fgr or Hck were then expressed in the cells, followed by assessment of inhibitor sensitivity. For these experiments, each cell population was incubated with A-419259 over the range of concentrations shown, and cell viability was assessed 48 h later using the Cell Titer Blue assay. Each data point was assessed in triplicate, and raw fluorescence values were normalized to the values observed in the absence of inhibitor for each cell population and are plotted as mean values ± SD. Inhibitor-response curves were best-fit by non-linear regression analysis (Graph Pad Prism, v.7), yielding IC_50_ values of 89.1 nM (control), 128.9 nM (+ Hck) and 173.3 nM (+ Fgr).
(PDF)

S10 Fig SYK expression is upregulated in A-419259-resistant AML cell populations. Heat map of relative mRNA expression levels in parent and inhibitor-resistant MV4-11, MOLM13, and MOLM14 cells as determined by qPCR of A-419259 target kinases identified by KINOMEscan analysis. Of the 27 kinases examined, only Syk expression was consistently increased in at least one resistant population from all three cell lines. Relative expression values were calculated as the base 2 antilog of the qPCR ΔCt values relative to GAPDH for each kinase. These values were then plotted as a distribution relative to the mean value for all 27 kinases analyzed in each sample. All determinations were made on at least three independent RNA samples from each cell line. For additional details about methods, see Weir, et al. *ACS Chem*. *Biol*. 23:1551, 2018; PMID: 29763550.
(PDF)

S11 Fig Inhibition of Syk kinase activity does not affect resistance to A-419259. These experiments used the Syk-selective inhibitor PRT062607 (P505-15) to probe the role of Syk kinase activity in acquired resistance to A-419259 in our inhibitor-resistant AML cell populations. In an initial experiment, the IC_50_ values for growth suppression was determined for parent and resistant cells using the CellTiter-Blue assay (values in Table at right). Based on these results, we then tested the effect of submaximal concentrations PRT062607 on A-419259 inhibitory activity in each cell population. Each population was treated with 0 μM (black), 50 μM (blue), 100 μM (green) or 200 μM (red) PRT062607 over a range of A-419259 concentrations as shown in the plots below. Cell viability was determined 72 h later using the Cell-titer Blue assay. Each value was normalized to the no-drug control, and the resulting concentration-response curves were generated by non-linear curve fitting (Prism v7.0). If Syk over-expression and activity contribute to A-419259 resistance, then addition of the Syk inhibitor would be predicted to shift the A-419259 concentration-response curve to left (re-sensitization) in the resistant populations. However, no significant PRT062607-dependent shifts were observed, suggesting that Syk does not contribute to the A-419259-resistant phenotype.
(PDF)

S1 Table Complete KINOMEscan dataset for A-419259. KINOMEscan values represent the percent of residual target kinase interaction with the immobilized probe compound at a A-419259 test concentration of 1.0 μM relative to control wells that contain DMSO. Therefore, a value of 0% control equals 100% probe displacement while a value of 100% control equals no binding of A-419259 to the target kinase. KINOMEscan profiling was performed by DiscoverX, which is now part of Eurofins.
(PDF)
