# Supplementary material for: Expression of myeloid Src-family kinases is associated with poor prognosis in AML and influences Flt3-ITD kinase inhibitor acquired resistance
Source: PLoS One. 2019 Dec 2;14(12):e0225887. doi: 10.1371/journal.pone.0225887 (PMC6886798; doi:10.1371/journal.pone.0225887)
Supplement: S1 Fig — (A) Hck, Fgr, and Lyn transcript levels are shown as the number of kinase cDNA fragments per kilobase of transcript per million mapped reads for all AML patients in the TCGA cohort (n = 163). Dots represent individual patient expression data, with the dot color representing Flt3 mutational status (grey, wild type; red, ITD; blue, D835Y). The plots compare Fgr vs. Hck (left), Lyn vs. Hck (middle) and Lyn vs. Fgr (right). Shown below each plot is the Pearson correlation coefficient (r) and p-value for each comparison. Code used to generate these plots is available on GitHub as described under Materials and Methods. (B) Distribution of Hck, Fgr, and Lyn expression levels in the presence of common AML mutations, including FLT3, NPM1, IDH1/2, DNMT3A, RUNX1, p53, NRAS, CEBPA, WT1, and PTPN11. Kinase transcript levels are shown as box and whisker plots, where the box defines the 25-75th percentile, the vertical bar is the median value, and the whiskers show the 5th to 95th percentiles. Each gene symbol includes all mutations present within that locus, with the exception of Flt3-ITD and Flt3-D835Y which are specific mutations. RUNX1T1 is the RUNX1-RUNX1T1 translocation associated with AML. (PDF) [file pone.0225887.s001.pdf]

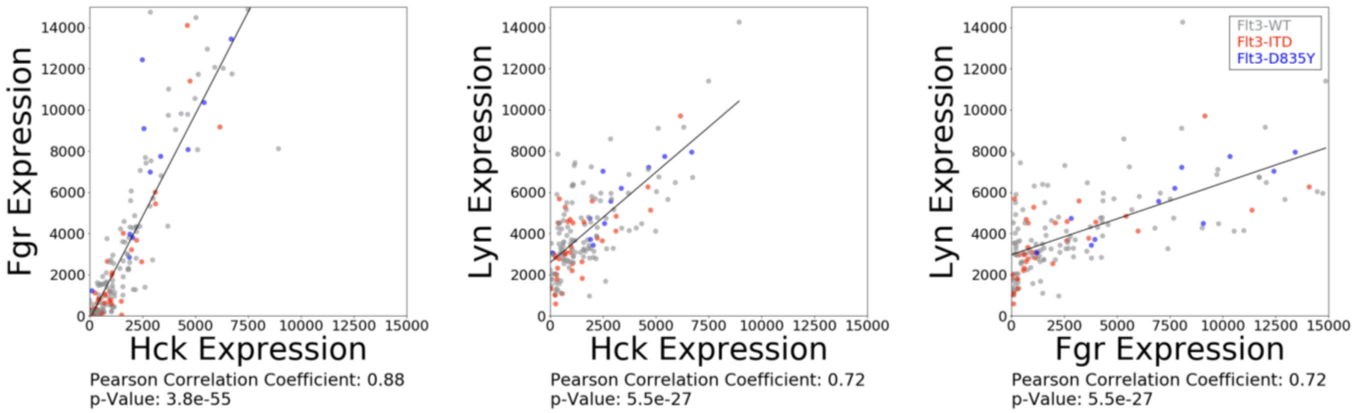

Figure S1. Pairwise correlation analysis of Hck, Fgr and Lyn transcript levels across all AML samples in the TCGA cohort. Hck, Fgr, and Lyn transcript levels are shown as the number of kinase cDNA fragments per kilobase of transcript per million mapped reads for all AML patients in the TCGA cohort (n=163). Dots represent individual patient expression data, with the dot color representing Flt3 mutational status (grey, wild type; red, ITD; blue, D835Y). The plots compare Fgr vs. Hck (*left*), Lyn vs. Hck (*middle*) and Lyn vs. Fgr (*right*). Shown below each plot is the Pearson correlation coefficient and p-value for each comparison. Code used to generate these plots is available on GitHub as described under Materials and Methods.
