# Supplementary material for: Expression of myeloid Src-family kinases is associated with poor prognosis in AML and influences Flt3-ITD kinase inhibitor acquired resistance
Source: PLoS One. 2019 Dec 2;14(12):e0225887. doi: 10.1371/journal.pone.0225887 (PMC6886798; doi:10.1371/journal.pone.0225887)
Supplement: S2 Fig — Transcript levels for Hck, Fgr and Lyn were downloaded for all tumors available on cBioPortal from the TCGA database. Data are shown as the number of cDNA fragments per kilobase of transcript per million mapped reads (FKPM). Each box and whisker plot shows the median value (middle line in box), 25th-75th percentiles (edges of box), and outliers (whiskers) for each data set. Code used to generate these plots is available on GitHub as described under Materials and Methods. (PDF) [file pone.0225887.s002.pdf]

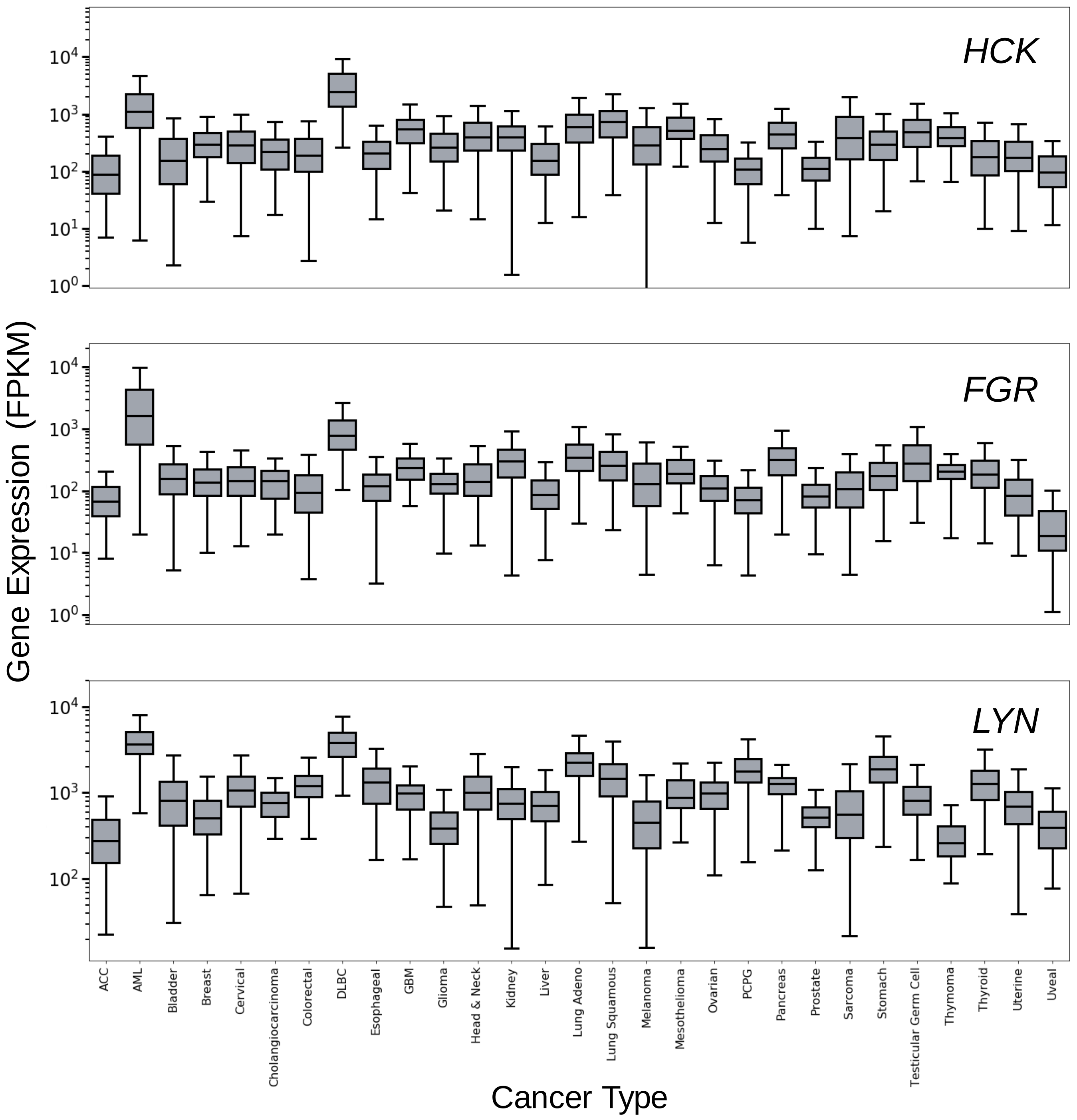

Figure S2. Comparison of Hck, Fgr and Lyn transcript levels across all tumors in the TCGA cohort. Transcript levels for Hck, Fgr and Lyn were downloaded for all tumors available on cBioPortal from the TCGA database. Data are shown as the number of cDNA fragments per kilobase of transcript per million mapped reads (FKPM). Each box and whisker plot shows the median value (middle line in box), 25th-75th percentiles (edges of box), and outliers (whiskers) for each data set. Code used to generate these plots is available on GitHub as described under Materials and Methods.
