# Supplementary material for: Expression of myeloid Src-family kinases is associated with poor prognosis in AML and influences Flt3-ITD kinase inhibitor acquired resistance
Source: PLoS One. 2019 Dec 2;14(12):e0225887. doi: 10.1371/journal.pone.0225887 (PMC6886798; doi:10.1371/journal.pone.0225887)
Supplement: S3 Fig — Each of the TF-1/Flt3-ITD cell populations indicated at the top (wild-type; D835Y; F691L ± wild-type Hck or Fgr) were treated with A-419259 at the nM concentrations shown or with 0.1% DMSO (carrier solvent) as control. Following overnight incubation, Flt3 was immunoprecipitated and analyzed for phosphotyrosine content by immunoblotting. Anti-phosphotyrosine immunoreactivity was detected using the Odyssey infrared imaging system. Positions of molecular weight markers (M) are shown in kDa. The phosphorylated Flt3-ITD bands are indicated by the arrows; in some cases, a lower molecular weight Flt3 band is observed which corresponds to the unglycosylated form of the receptor. Control blots were performed with anti-Flt3 antibodies to verify kinase recovery (lower panel in each set); the major Flt3 species recovered is the unglycosylated form (arrows). Band intensities for phosphorylated Flt3 were normalized to the Flt3 protein levels from at least three 3 independent experiments, and the ratios were used to generate the IC50 values shown in Fig 4. (PDF) [file pone.0225887.s003.pdf]

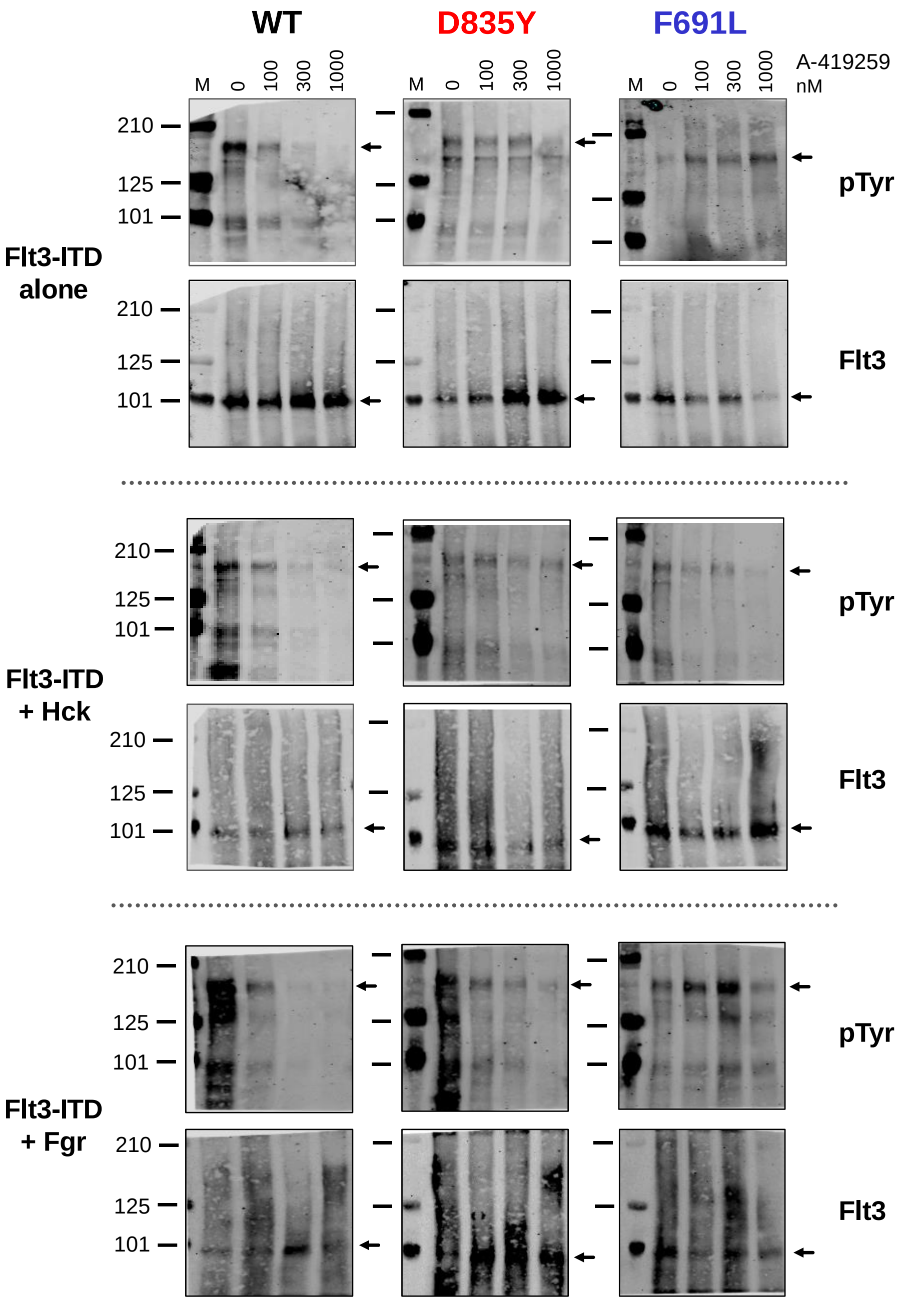

Figure S3. **Anti-phosphotyrosine immunoblots from TF-1 cells expressing Flt3-ITD alone or with Hck and Fgr.** Each of the TF-1/Flt3-ITD cell populations indicated at the top (wild-type; D835Y; F691L ± wild-type Hck or Fgr) were treated with A-419259 at the nM concentrations shown or with 0.1% DMSO (carrier solvent) as control. Following overnight incubation, Flt3 was immunoprecipitated and analyzed for phosphotyrosine content by immunoblotting. Anti-phosphotyrosine immunoreactivity was detected using the Odyssey infrared imaging system. Positions of molecular weight markers (M) are shown in kDa. The phosphorylated Flt3-ITD bands are indicated by the arrows; in some cases, a lower molecular weight Flt3 band is observed which corresponds to the unglycosylated form of the receptor. Control blots were performed with anti-Flt3 antibodies to verify kinase recovery (lower panel in each set); the major Flt3 species recovered is the unglycosylated form (*arrows*). Band intensities for phosphorylated Flt3 were normalized to the Flt3 protein levels from 3 or 4 independent experiments, and the mean ratios are presented in main Figure 4.
