# Supplementary material for: Expression of myeloid Src-family kinases is associated with poor prognosis in AML and influences Flt3-ITD kinase inhibitor acquired resistance
Source: PLoS One. 2019 Dec 2;14(12):e0225887. doi: 10.1371/journal.pone.0225887 (PMC6886798; doi:10.1371/journal.pone.0225887)
Supplement: S4 Fig — Recombinant near-full-length kinases, consisting of the SH2, SH3 and kinase domains plus the negative regulatory tail, were expressed in E. coli in the presence of Csk (to phosphorylate the tail tyrosine) and PTP1B (to keep the activation loop dephosphorylated). Purified kinases were assayed in vitro using the Z’-LYTE kinase assay (ThermoFisher) and the Tyr-2 peptide substrate (final concentration of 1.0 μM). A) Determination of Km values for ATP. Kinase activity was determined over the range of ATP concentrations shown. Reaction velocities were determined by quenching each reaction at various time points. The resulting curves were fit to the Michaelis-Menten equation using GraphPad Prism v7.04, and the resulting Km values are shown in the Table at right. B) Determination of intrinsic kinase activity. Each kinase was assayed over a range of input amounts with the ATP concentrations set to the Km. Kinase titration curves were best-fit by non-linear regression analysis (Prism) and the resulting EC50 values are shown in in the table. Kinase forms color-coded as per the Table are also used in the plots in part A and B. (PDF) [file pone.0225887.s004.pdf]

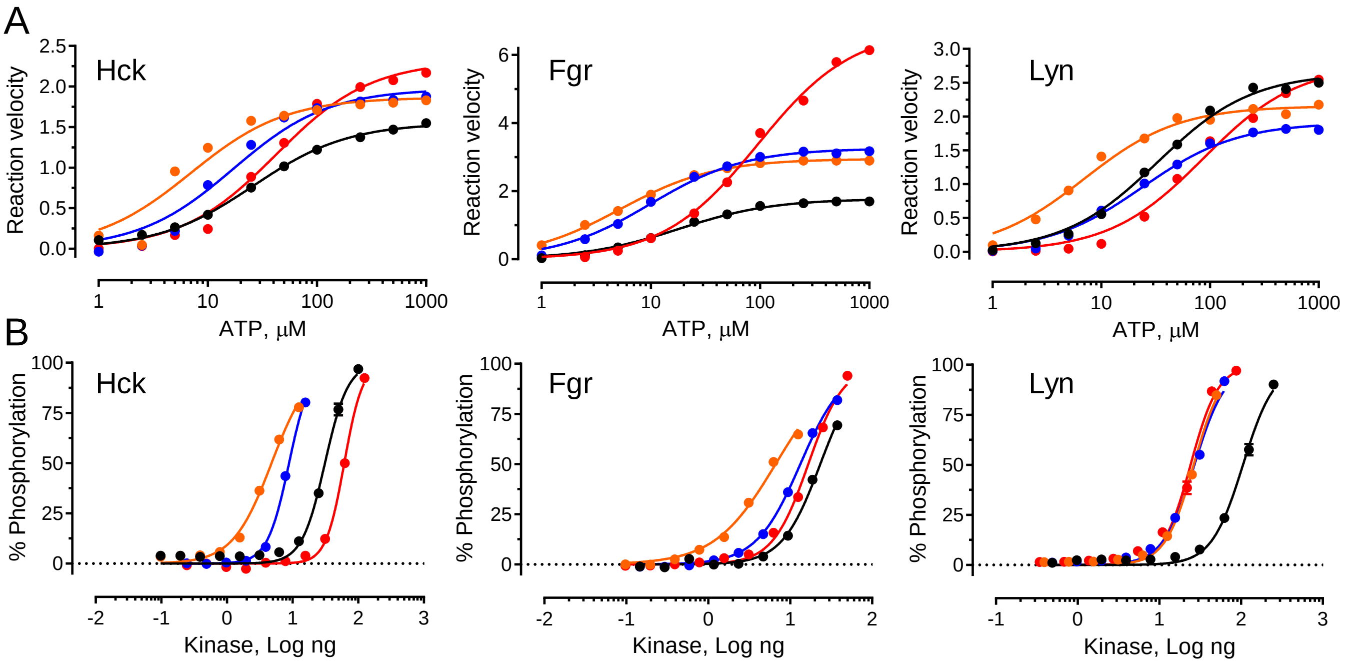

Figure S4. ***In vitro*** **kinetics of wild-type and gatekeeper mutants of Hck, Fgr and Lyn.** Recombinant near-full-length kinases, consisting of the SH2, SH3 and kinase domains plus the negative regulatory tail, were expressed in *E. coli* in the presence of Csk (to phosphorylate the tail tyrosine) and PTP1B (to keep the activation loop dephosphorylated). Purified kinases were assayed *in vitro* using the Z'-LYTE kinase assay (ThermoFisher) and the Tyr-2 peptide substrate (final concentration of 1.0 µM). **A**) Determination of K_m values for ATP. Kinase activity was determined over the range of ATP concentrations shown. Reaction velocities were determined by quenching each reaction at various time points. The resulting curves were fit to the Michaelis-Menten equation using GraphPad Prism v7.04, and the resulting K_m values are shown in the Table at right. **B**) Determination of intrinsic kinase activity. Each kinase was assayed over a range of input amounts with the ATP concentrations set to the K_m. Kinase titration curves were best-fit by non-linear regression analysis (Prism) and the resulting EC_{50} values are shown in in the table. Kinase forms color-coded as per the Table are also used in the plots in part A and B.

Kinase	Form	ATP K_m, µM	Kinase EC_{50}, ng
Hck	**Wild-type**	26.0	31.1
	**T338M**	6.9	4.8
	**T338L**	17.2	8.9
	**T338F**	42.0	61.4
Fgr	**Wild-type**	17.9	23.3
	**T338M**	5.3	6.6
	**T338L**	10.0	13.3
	**T338F**	95.7	16.6
Lyn	**Wild-type**	32.8	105.7
	**T338M**	7.0	26.4
	**T338L**	24.0	26.9
	**T338F**	87.3	24.1
