# Supplementary material for: Expression of myeloid Src-family kinases is associated with poor prognosis in AML and influences Flt3-ITD kinase inhibitor acquired resistance
Source: PLoS One. 2019 Dec 2;14(12):e0225887. doi: 10.1371/journal.pone.0225887 (PMC6886798; doi:10.1371/journal.pone.0225887)
Supplement: S5 Fig — Wild-type and gatekeeper mutants of Fgr and Hck were stably expressed in TF-1 cells. After selection with G418, cells were cultured in the presence or absence of GM-CSF and viability was monitored daily using the CellTiter Blue assay (Promega). Data are presented as relative fluorescence units, which increase as a function of cell proliferation. TF-1 cells transformed with Flt3-ITD served as a positive control, while cells transduced with an empty vector served as negative control. Expression of each kinase was confirmed by immunoblotting (data not shown). TF-1 cells expressing Fgr-T338M showed GM-CSF-independent proliferation to the same extent as Flt3-ITD, while Fgr-T338F produced a partial cytokine-independent phenotype. (PDF) [file pone.0225887.s005.pdf]

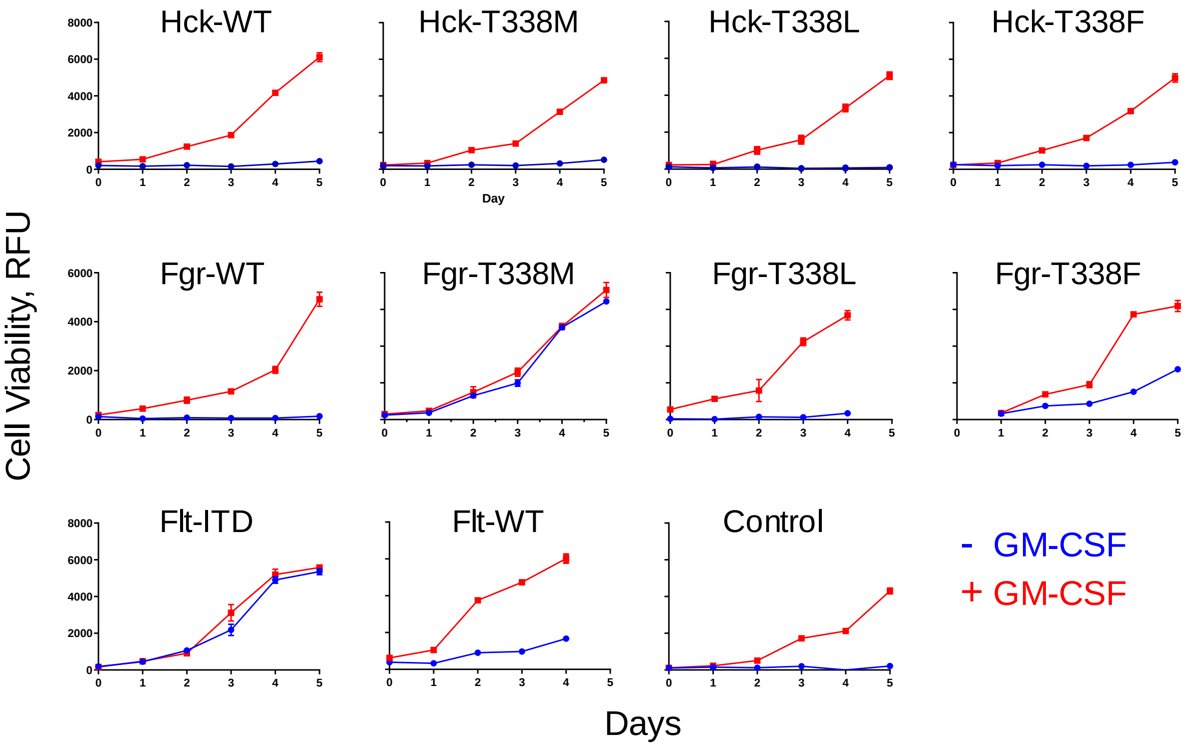

Figure S5. Fgr but not Hck gatekeeper mutants transform TF-1 myeloid cells to cytokine-independent growth. Wild-type and gatekeeper mutants of Fgr and Hck were stably expressed in TF-1 cells. After selection with G418, cells were cultured in the presence or absence of GM-CSF and viability was monitored daily using the CellTiter Blue assay (Promega). Data are presented as relative fluorescence units, which increase as a function of cell proliferation. TF-1 cells transformed with Flt3-ITD served as a positive control, while cells transduced with an empty vector served as negative control. Expression of each kinase was confirmed by immunoblotting (*data not shown*). TF-1 cells expressing Fgr-T338M showed GM-CSF-independent proliferation to the same extent as Flt3-ITD, while Fgr-T338F produced a partial cytokine-independent phenotype.
