# Supplementary material for: Expression of myeloid Src-family kinases is associated with poor prognosis in AML and influences Flt3-ITD kinase inhibitor acquired resistance
Source: PLoS One. 2019 Dec 2;14(12):e0225887. doi: 10.1371/journal.pone.0225887 (PMC6886798; doi:10.1371/journal.pone.0225887)
Supplement: S6 Fig — Wild-type and Thr338 gatekeeper mutants of Hck and Fgr were expressed in TF-1/Flt3-ITD cells using recombinant retroviruses with a second selection marker (see Materials and methods). Each cell population was then plated at equal density, and cell viability was assessed 48 h later using the Cell Titer Blue assay. Raw fluorescence values were normalized to those obtained with control TF-1/Flt3-ITD cells. Each population was assayed in triplicate, and the average values are shown ± SEM. End-point proliferation varied by less than 15% relative to the control TF-1/Flt3-ITD cells, with the exception of the cells expressing the Hck and Fgr T338F mutants, which showed increases of 24% and 54%, respectively (p < 0.0001 by Student’s t-test in each case). (PDF) [file pone.0225887.s006.pdf]

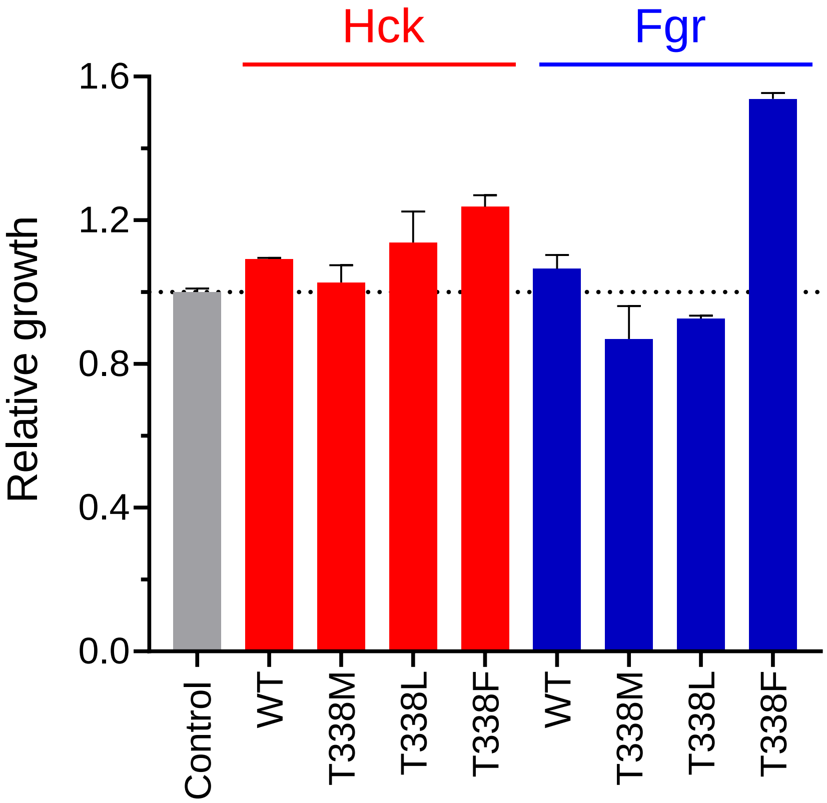

Figure S6. Expression of Hck and Fgr gatekeeper mutants does not markedly affect proliferation of TF-1/Flt3-ITD cells. Wild-type and Thr338 gatekeeper mutants of Hck and Fgr were expressed in TF-1/Flt3-ITD cells using recombinant retroviruses with a second selection marker (see Materials and Methods). Each cell population was then plated at equal density, and cell viability was assessed 48 h later using the Cell Titer Blue assay. Raw fluorescence values were normalized to those obtained with control TF-1/Flt3-ITD cells. Each population was assayed in triplicate, and the average values are shown ± SEM. End-point proliferation varied by less than 15% relative to the control TF-1/Flt3-ITD cells, with the exception of the cells expressing the Hck and Fgr T338F mutants, which showed increases of 24% and 54%, respectively ($p < 0.0001$ by Student's t-test in each case).
