# Supplementary material for: Expression of myeloid Src-family kinases is associated with poor prognosis in AML and influences Flt3-ITD kinase inhibitor acquired resistance
Source: PLoS One. 2019 Dec 2;14(12):e0225887. doi: 10.1371/journal.pone.0225887 (PMC6886798; doi:10.1371/journal.pone.0225887)
Supplement: S7 Fig — TF-1 cells co-expressing Flt3-ITD together with wild-type and gatekeeper mutants of Hck (upper panels) or Fgr (lower panels) were incubated overnight with A-419259 at the nM concentrations shown. Hck and Fgr were immunoprecipitated from clarified cell extracts and immunoblotted for activation loop phosphorylation as a marker for kinase activity (pY416; upper panels in each set) as well as kinase protein recovery (lower panels in each set). Kinase protein and pY416 immunoreactivity were quantified directly using the Odyssey infrared imaging system. Representative uncropped pY416 and kinase protein immunoblot images are shown, along with molecular weight markers in kDa (M). For the pY416 blots, the positions of the pY416 bands are indicated by the arrows (pHck and pFgr); in most cases, the heavy chains of the anti-Hck and anti-Fgr antibodies used to immunoprecipitate the kinases are also observed (IgH; grey arrows). Replicate blots were used to generate the IC50 values shown in Fig 7. (PDF) [file pone.0225887.s007.pdf]

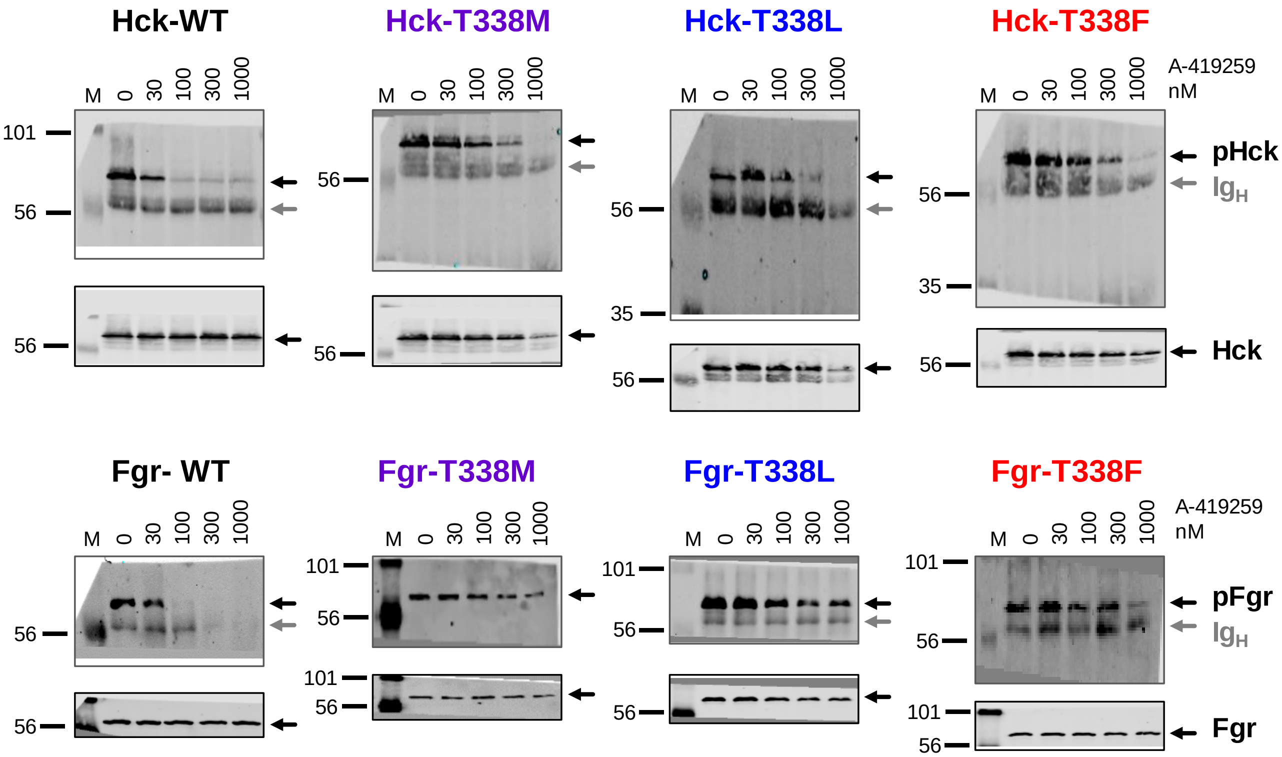

Figure S7. Immunoblots of activation loop tyrosine phosphorylation of Hck and Fgr gatekeeper mutants in A-419259-treated TF-1 cells. TF-1 cells co-expressing Flt3-ITD together with wild-type and gatekeeper mutants of Hck (*upper panels*) or Fgr (*lower panels*) were incubated overnight with A-419259 at the nM concentrations shown. Hck and Fgr were immunoprecipitated from clarified cell extracts and immunoblotted for activation loop phosphorylation as a marker for kinase activity (pY416; upper panels in each set) as well as kinase protein recovery (lower panels in each set). Kinase protein and pY416 immunoreactivity were quantified directly using the Odyssey infrared imaging system. Representative uncropped pY416 and kinase protein immunoblot images are shown, along with molecular weight markers in kDa (*M*). For the pY416 blots, the positions of the pY416 bands are indicated by the arrows (pHck and pFgr); in most cases, the heavy chains of the anti-Hck and anti-Fgr antibodies used to immunoprecipitate the kinases are also observed (*Ig_H; grey arrows*).
