# Supplementary material for: Expression of myeloid Src-family kinases is associated with poor prognosis in AML and influences Flt3-ITD kinase inhibitor acquired resistance
Source: PLoS One. 2019 Dec 2;14(12):e0225887. doi: 10.1371/journal.pone.0225887 (PMC6886798; doi:10.1371/journal.pone.0225887)
Supplement: S8 Fig — Relative expression of A-419259 target kinases (based on KINOMEscan profiling) was determined in a cohort of 26 AML patient bone marrow samples using quantitative real-time RT-PCR (qPCR). Relative expression values are represented as box-and-whisker plots, with statistical outliers (value > 3σ) represented as grey diamonds. A description of these patient samples and the methods used to determine the relative kinase expression profiles is reported in detail in Weir, et al. ACS Chem. Biol. 23:1551, 2018; PMID: 29763550. Kinase expression profiles in the three AML cell lines were determined using the same qPCR approach, and these values are overlaid on the patient data using the color-coded dots as indicated. Code used to generate these plots is available on GitHub as described under Materials and Methods. (PDF) [file pone.0225887.s008.pdf]

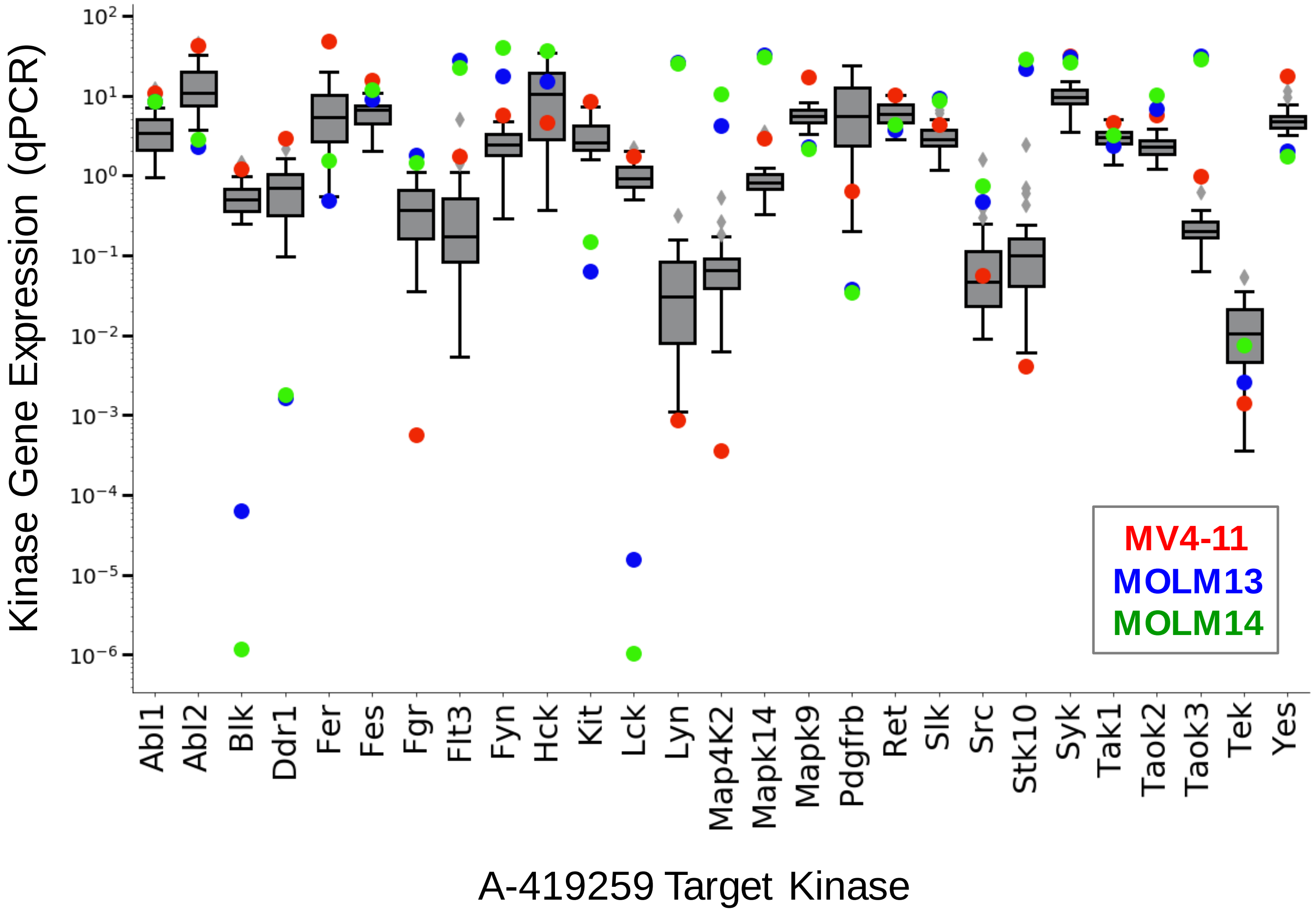

Figure S8. The Flt3-ITD⁺ AML cell lines MV4-11, MOLM13 and MOLM14 express similar levels of A-419259 target kinases as AML patient cells. Relative expression of A-419259 target kinases (based on KINOMEscan profiling) was determined in a cohort of 26 AML patient bone marrow samples using quantitative real-time RT-PCR (qPCR). Relative expression values are represented as box-and-whisker plots, with statistical outliers (value > 3σ) represented as grey diamonds. A description of these patient samples and the methods used to determine the relative kinase expression profiles is reported in detail in Weir, *et al*. *ACS Chem. Biol*. 23:1551, 2018; PMID: 29763550. Kinase expression profiles in the three AML cell lines were determined using the same qPCR approach, and these values are overlaid on the patient data using the color-coded dots as indicated. Code used to generate these plots is available on GitHub as described under Materials and Methods.
