# Supplementary material for: Expression of myeloid Src-family kinases is associated with poor prognosis in AML and influences Flt3-ITD kinase inhibitor acquired resistance
Source: PLoS One. 2019 Dec 2;14(12):e0225887. doi: 10.1371/journal.pone.0225887 (PMC6886798; doi:10.1371/journal.pone.0225887)
Supplement: S9 Fig — TF-1 cells were transformed to GM-CSF independence by expression of the N676S mutant of Flt3-ITD. Wild-type Fgr or Hck were then expressed in the cells, followed by assessment of inhibitor sensitivity. For these experiments, each cell population was incubated with A-419259 over the range of concentrations shown, and cell viability was assessed 48 h later using the Cell Titer Blue assay. Each data point was assessed in triplicate, and raw fluorescence values were normalized to the values observed in the absence of inhibitor for each cell population and are plotted as mean values ± SD. Inhibitor-response curves were best-fit by non-linear regression analysis (Graph Pad Prism, v.7), yielding IC50 values of 89.1 nM (control), 128.9 nM (+ Hck) and 173.3 nM (+ Fgr). (PDF) [file pone.0225887.s009.pdf]

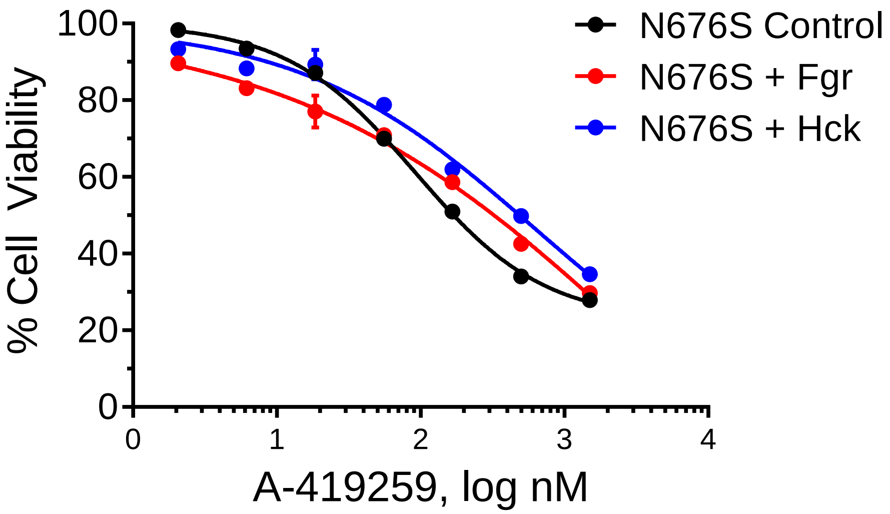

Figure S9. Expression of Hck or Fgr does not influence sensitivity of TF-1/Flt3-ITD N676S cells to A-419259. TF-1 cells were transformed to GM-CSF independence by expression of the N676S mutant of Flt3-ITD. Wild-type Fgr or Hck were then expressed in the cells, followed by assessment of inhibitor sensitivity. For these experiments, each cell population was incubated with A-419259 over the range of concentrations shown, and cell viability was assessed 48 h later using the Cell Titer Blue assay. Each data point was assessed in triplicate, and raw fluorescence values were normalized to the values observed in the absence of inhibitor for each cell population and are plotted as mean values ± SD. Inhibitor-response curves were best-fit by non-linear regression analysis (Graph Pad Prism, v.7), yielding IC_{50} values of 89.1 nM (control), 128.9 nM (+ Hck) and 173.3 nM (+ Fgr).
