# Supplementary material for: Expression of myeloid Src-family kinases is associated with poor prognosis in AML and influences Flt3-ITD kinase inhibitor acquired resistance
Source: PLoS One. 2019 Dec 2;14(12):e0225887. doi: 10.1371/journal.pone.0225887 (PMC6886798; doi:10.1371/journal.pone.0225887)
Supplement: S10 Fig — Heat map of relative mRNA expression levels in parent and inhibitor-resistant MV4-11, MOLM13, and MOLM14 cells as determined by qPCR of A-419259 target kinases identified by KINOMEscan analysis. Of the 27 kinases examined, only Syk expression was consistently increased in at least one resistant population from all three cell lines. Relative expression values were calculated as the base 2 antilog of the qPCR ΔCt values relative to GAPDH for each kinase. These values were then plotted as a distribution relative to the mean value for all 27 kinases analyzed in each sample. All determinations were made on at least three independent RNA samples from each cell line. For additional details about methods, see Weir, et al. ACS Chem. Biol. 23:1551, 2018; PMID: 29763550. (PDF) [file pone.0225887.s010.pdf]

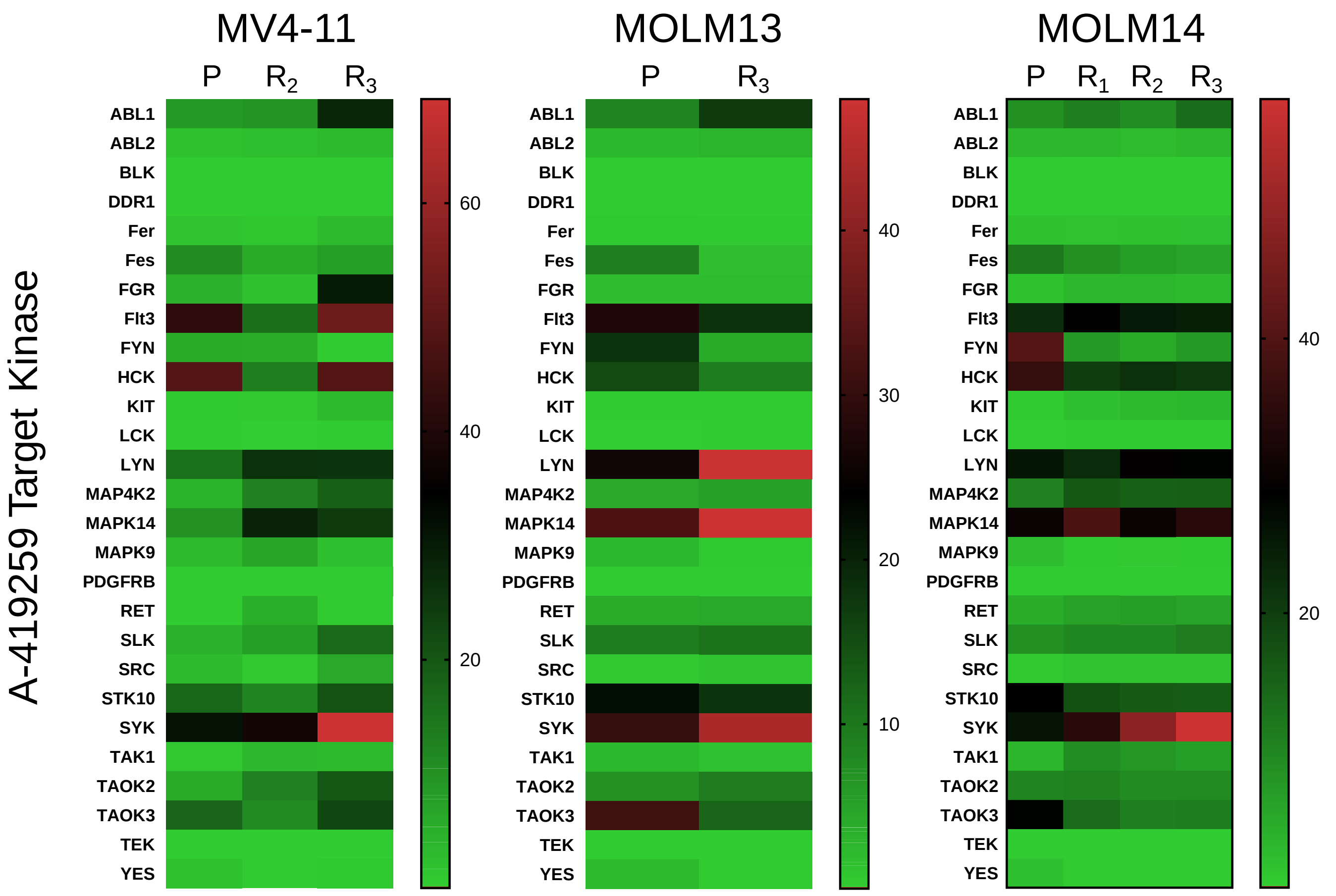

Figure S10. SYK expression is upregulated in A-419259 resistant AML cell populations. Heat map of relative mRNA expression levels in parent and inhibitor-resistant MV4-11, MOLM13, and MOLM14 cells as determined by qPCR of A-419259 target kinases identified by KINOMEscan analysis. Of the 27 kinases examined, only Syk expression was consistently increased in at least one resistant population from all three cell lines. Relative expression values were calculated as the base 2 antilog of the qPCR ΔCt values relative to GAPDH for each kinase. These values were then plotted as a distribution relative to the mean value for all 27 kinases analyzed in each sample. All determinations were made on at least three independent RNA samples from each cell line. For additional details about methods, see Weir, et al. *ACS Chem. Biol*. 23:1551, 2018; PMID: 29763550.
