# Supplementary material for: Expression of myeloid Src-family kinases is associated with poor prognosis in AML and influences Flt3-ITD kinase inhibitor acquired resistance
Source: PLoS One. 2019 Dec 2;14(12):e0225887. doi: 10.1371/journal.pone.0225887 (PMC6886798; doi:10.1371/journal.pone.0225887)

Figure S11. Inhibition of Syk kinase activity does not affect resistance to A-419259. These experiments used the Syk-selective inhibitor PRT062607 (P505-15) to probe the role of Syk kinase activity in acquired resistance to A-419259 in our inhibitor-resistant AML cell populations. In an initial experiment, the IC_{50} values for growth suppression was determined for parent and resistant cells using the CellTiter-Blue assay (values in Table at right). Based on these results, we then tested the effect of submaximal concentrations PRT062607 on A-419259 inhibitory activity in each cell population. Each population was treated with 0 µM (black), 50 µM (blue), 100 µM (green) or 200 µM (red) PRT062607 over a range of A-419259 concentrations as shown in the plots below. Cell viability was determined 72 h later using the Cell-titer Blue assay. Each value was normalized to the no-drug control, and the resulting concentration-response curves were generated by non-linear curve fitting (Prism v7.0). If Syk over-expression and activity contribute to A-419259 resistance, then addition of the Syk inhibitor would be predicted to shift the A-419259 concentration-response curve to left (re-sensitization) in the resistant populations. However, no significant PRT062607-dependent shifts were observed, suggesting that Syk does not contribute to the A-419259-resistant phenotype.

Cell Line	Population	PRT062607 IC_{50}, nM
MV4-11	Parent	453.2 ± 90.7
	R_2	1717.0 ± 93.5
	R_3	794.6 ± 128.4
MOLM13	Parent	1377.7 ± 123.6
	R_3	1991.0 ± 377.4
MOLM14	Parent	1328.4 ± 183.1
	R_1	2033.5 ± 1085.4
	R_2	1644.7 ± 153.6
	R_3	1195.7 ± 128.0

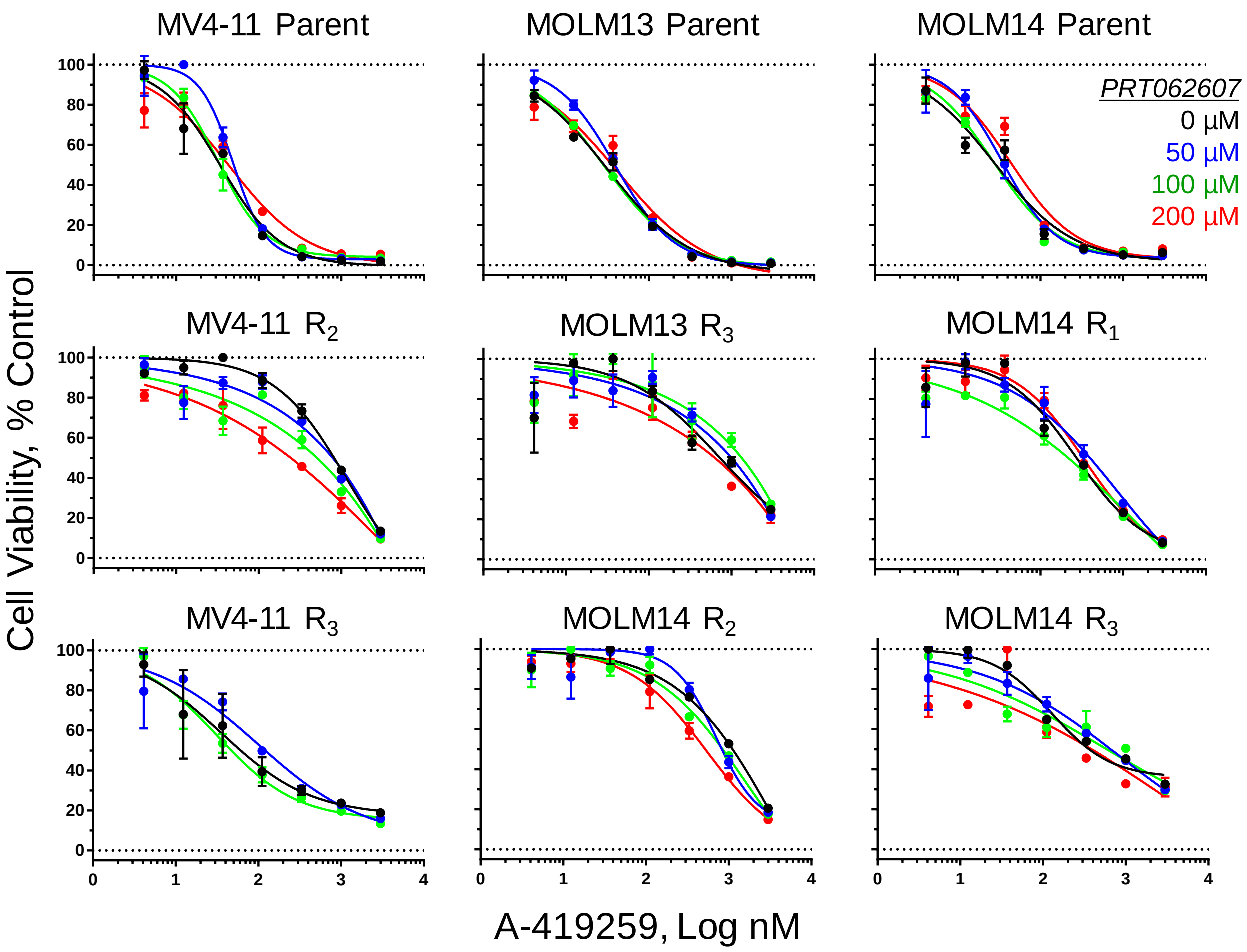

Supplement: S11 Fig — These experiments used the Syk-selective inhibitor PRT062607 (P505-15) to probe the role of Syk kinase activity in acquired resistance to A-419259 in our inhibitor-resistant AML cell populations. In an initial experiment, the IC50 values for growth suppression was determined for parent and resistant cells using the CellTiter-Blue assay (values in Table at right). Based on these results, we then tested the effect of submaximal concentrations PRT062607 on A-419259 inhibitory activity in each cell population. Each population was treated with 0 μM (black), 50 μM (blue), 100 μM (green) or 200 μM (red) PRT062607 over a range of A-419259 concentrations as shown in the plots below. Cell viability was determined 72 h later using the Cell-titer Blue assay. Each value was normalized to the no-drug control, and the resulting concentration-response curves were generated by non-linear curve fitting (Prism v7.0). If Syk over-expression and activity contribute to A-419259 resistance, then addition of the Syk inhibitor would be predicted to shift the A-419259 concentration-response curve to left (re-sensitization) in the resistant populations. However, no significant PRT062607-dependent shifts were observed, suggesting that Syk does not contribute to the A-419259-resistant phenotype. (PDF) [file pone.0225887.s011.pdf]
