# Supplementary material for: Expression of myeloid Src-family kinases is associated with poor prognosis in AML and influences Flt3-ITD kinase inhibitor acquired resistance
Source: PLoS One. 2019 Dec 2;14(12):e0225887. doi: 10.1371/journal.pone.0225887 (PMC6886798; doi:10.1371/journal.pone.0225887)
Supplement: S1 Table — KINOMEscan values represent the percent of residual target kinase interaction with the immobilized probe compound at a A-419259 test concentration of 1.0 μM relative to control wells that contain DMSO. Therefore, a value of 0% control equals 100% probe displacement while a value of 100% control equals no binding of A-419259 to the target kinase. KINOMEscan profiling was performed by DiscoverX, which is now part of Eurofins. (PDF) [file pone.0225887.s012.pdf]

Table S1. Complete KINOMEscan dataset for A-419259. KINOMEscan values represent the percent of residual target kinase interaction with the immobilized probe compound at a A-419259 test concentration of 1.0 µM relative to control wells that contain DMSO. Therefore, a value of 0% control equals 100% probe displacement while a value of 100% control equals no binding of A-419259 to the target kinase. KINOMEscan profiling was performed by DiscoverX, which is now part of Eurofins.

KINOMEscan gene symbol	% Control
AAK1	100
ABL1(E255K)-phosphorylated	0.5
ABL1(F317I)-nonphosphorylated	2.5
ABL1(F317I)-phosphorylated	2.4
ABL1(F317L)-nonphosphorylated	4.7
ABL1(F317L)-phosphorylated	0
ABL1(H396P)-nonphosphorylated	0.2
ABL1(H396P)-phosphorylated	0.65
ABL1(M351T)-phosphorylated	1.6
ABL1(Q252H)-nonphosphorylated	0.85
ABL1(Q252H)-phosphorylated	0.5
ABL1(T315I)-nonphosphorylated	41
ABL1(T315I)-phosphorylated	83
ABL1(Y253F)-phosphorylated	0.35
ABL1-nonphosphorylated	0.1
ABL1-phosphorylated	0.25
ABL2	4.9
ACVR1	100
ACVR1B	100
ACVR2A	100
ACVR2B	100
ACVRL1	100
ADCK3	100
ADCK4	100
AKT1	100
AKT2	92
AKT3	100
ALK	29
ALK(C1156Y)	21
ALK(L1196M)	68
AMPK-alpha1	43
AMPK-alpha2	42
ANKK1	73
ARK5	90
ASK1	100
ASK2	62
AURKA	36
AURKB	100
AURKC	79
AXL	6.1
BIKE	80
BLK	0.05
BMPR1A	100
BMPR1B	92
BMPR2	97
BMX	30
BRAF	66
BRAF(V600E)	45
BRK	0.2
BRSK1	14
BRSK2	10
BTK	0.1
BUB1	100
CAMK1	92
CAMK1B	100
CAMK1D	97
CAMK1G	100
CAMK2A	88
CAMK2B	100
CAMK2D	100
CAMK2G	100
CAMK4	100
CAMKK1	100
CAMKK2	100
CASK	67
CDC2L1	61
CDC2L2	91
CDC2L5	77
CDK11	3.9
CDK2	70
CDK3	99
CDK4	91
CDK4-cyclinD1	27
CDK4-cyclinD3	81
CDK5	83
CDK7	28
CDK8	28
CDK9	65
CDKL1	99

CDKL2	70
CDKL3	100
CDKL5	100
CHEK1	54
CHEK2	40
CIT	79
CLK1	100
CLK2	100
CLK3	100
CLK4	100
CSF1R	5.8
CSF1R-autoinhibited	57
CSK	2.1
CSNK1A1	20
CSNK1A1L	64
CSNK1D	8.3
CSNK1E	1.1
CSNK1G1	80
CSNK1G2	84
CSNK1G3	100
CSNK2A1	100
CSNK2A2	66
CTK	89
DAPK1	99
DAPK2	98
DAPK3	100
DCAMKL1	43
DCAMKL2	87
DCAMKL3	100
DDR1	65
DDR2	59
DLK	54
DMPK	100
DMPK2	36
DRAK1	65
DRAK2	93
DYRK1A	99
DYRK1B	21
DYRK2	100
EGFR	26
EGFR(E746-A750del)	20
EGFR(G719C)	4.8
EGFR(G719S)	41
EGFR(L747-E749del, A750P)	57
EGFR(L747-S752del, P753S)	22

EGFR(L747-T751del,Sins)	16
EGFR(L858R)	9.7
EGFR(L858R,T790M)	29
EGFR(L861Q)	6
EGFR(S752-I759del)	6.7
EGFR(T790M)	0.2
EIF2AK1	99
EPHA1	58
EPHA2	100
EPHA3	95
EPHA4	100
EPHA5	80
EPHA6	94
EPHA7	100
EPHA8	72
EPHB1	96
EPHB2	100
EPHB3	100
EPHB4	100
EPHB6	6.8
ERBB2	2.7
ERBB3	0.2
ERBB4	0
ERK1	100
ERK2	77
ERK3	100
ERK4	56
ERK5	58
ERK8	100
ERN1	82
FAK	72
FER	100
FES	100
FGFR1	1.6
FGFR2	39
FGFR3	30
FGFR3(G697C)	27
FGFR4	62
FGR	0.85
FLT1	29
FLT3	0.65
FLT3(D835H)	12
FLT3(D835V)	0.15
FLT3(D835Y)	7.6
FLT3(ITD)	2.6

FLT3(ITD,D835V)	30
FLT3(ITD,F691L)	57
FLT3(K663Q)	5.6
FLT3(N841I)	5.3
FLT3(R834Q)	2.7
FLT3-autoinhibited	3.4
FLT4	19
FRK	5.3
FYN	5.1
GAK	69
GCN2(Kin.Dom.2,S808G)	79
GRK1	100
GRK2	100
GRK3	100
GRK4	100
GRK7	62
GSK3A	100
GSK3B	95
HASPIN	70
HCK	1.9
HIPK1	80
HIPK2	100
HIPK3	91
HIPK4	63
HPK1	44
HUNK	100
ICK	97
IGF1R	80
IKK-alpha	90
IKK-beta	90
IKK-epsilon	100
INSR	63
INSRR	86
IRAK1	98
IRAK3	89
IRAK4	90
ITK	14
JAK1(JH1domain-catalytic)	99
JAK1(JH2domain-pseudokinase)	100
JAK2(JH1domain-catalytic)	100
JAK3(JH1domain-catalytic)	65
JNK1	95
JNK2	94
JNK3	99
KIT	0.15

KIT(A829P)	5.9
KIT(D816H)	40
KIT(D816V)	70
KIT(L576P)	0
KIT(V559D)	0.05
KIT(V559D,T670I)	40
KIT(V559D,V654A)	18
KIT-autoinhibited	6.9
LATS1	30
LATS2	18
LCK	2.3
LIMK1	31
LIMK2	91
LKB1	100
LOK	20
LRRK2	98
LRRK2(G2019S)	100
LTK	36
LYN	1.7
LZK	85
MAK	100
MAP3K1	92
MAP3K15	79
MAP3K2	1.7
MAP3K3	6.3
MAP3K4	92
MAP4K2	98
MAP4K3	32
MAP4K4	83
MAP4K5	9.2
MAPKAPK2	100
MAPKAPK5	100
MARK1	82
MARK2	82
MARK3	83
MARK4	97
MAST1	64
MEK1	0.1
MEK2	0.2
MEK3	91
MEK4	89
MEK5	0.25
MEK6	98
MELK	21
MERTK	12

MET	58
MET(M1250T)	59
MET(Y1235D)	100
MINK	30
MKK7	46
MKNK1	63
MKNK2	8.3
MLCK	66
MLK1	88
MLK2	29
MLK3	100
MRCKA	98
MRCKB	100
MST1	34
MST1R	96
MST2	82
MST3	80
MST4	4.9
MTOR	90
MUSK	100
MYLK	94
MYLK2	95
MYLK4	96
MYO3A	2.7
MYO3B	17
NDR1	68
NDR2	80
NEK1	100
NEK10	100
NEK11	77
NEK2	81
NEK3	92
NEK4	100
NEK5	100
NEK6	65
NEK7	91
NEK9	90
NIK	100
NIM1	100
NLK	83
OSR1	100
p38-alpha	100
p38-beta	100
p38-delta	99
p38-gamma	100

PAK1	55
PAK2	91
PAK3	36
PAK4	93
PAK6	84
PAK7	89
PCTK1	99
PCTK2	59
PCTK3	100
PDGFRA	1.1
PDGFRB	0
PDPK1	100
PFCDPK1(P.falciparum)	0
PFPK5(P.falciparum)	78
PFTAIRE2	89
PFTK1	63
PHKG1	99
PHKG2	100
PIK3C2B	10
PIK3C2G	100
PIK3CA	100
PIK3CA(C420R)	100
PIK3CA(E542K)	85
PIK3CA(E545A)	100
PIK3CA(E545K)	95
PIK3CA(H1047L)	98
PIK3CA(H1047Y)	99
PIK3CA(I800L)	98
PIK3CA(M1043I)	100
PIK3CA(Q546K)	100
PIK3CB	64
PIK3CD	100
PIK3CG	61
PIK4CB	83
PIKFYVE	78
PIM1	100
PIM2	100
PIM3	100
PIP5K1A	100
PIP5K1C	89
PIP5K2B	94
PIP5K2C	95
PKAC-alpha	100
PKAC-beta	100
PKMYT1	100

PKN1	88
PKN2	100
PKNB(M.tuberculosis)	82
PLK1	100
PLK2	77
PLK3	72
PLK4	100
PRKCD	89
PRKCE	88
PRKCH	87
PRKCI	26
PRKCQ	66
PRKD1	63
PRKD2	63
PRKD3	17
PRKG1	100
PRKG2	100
PRKR	100
PRKX	100
PRP4	98
PYK2	88
QSK	93
RAF1	40
RET	0
RET(M918T)	0.1
RET(V804L)	24
RET(V804M)	21
RIOK1	77
RIOK2	100
RIOK3	64
RIPK1	100
RIPK2	3.6
RIPK4	84
RIPK5	7.1
ROCK1	90
ROCK2	62
ROS1	100
RPS6KA4(Kin.Dom.1-N-terminal)	66
RPS6KA4(Kin.Dom.2-C-terminal)	99
RPS6KA5(Kin.Dom.1-N-terminal)	77
RPS6KA5(Kin.Dom.2-C-terminal)	96
RSK1(Kin.Dom.1-N-terminal)	8.4
RSK1(Kin.Dom.2-C-terminal)	84
RSK2(Kin.Dom.1-N-terminal)	16
RSK2(Kin.Dom.2-C-terminal)	100

RSK3(Kin.Dom.1-N-terminal)	22
RSK3(Kin.Dom.2-C-terminal)	100
RSK4(Kin.Dom.1-N-terminal)	68
RSK4(Kin.Dom.2-C-terminal)	94
S6K1	27
SBK1	96
SGK	75
SgK110	100
SGK2	90
SGK3	77
SIK	2.1
SIK2	19
SLK	100
SNARK	26
SNRK	94
SRC	0.1
SRMS	0
SRPK1	100
SRPK2	97
SRPK3	99
STK16	100
STK33	30
STK35	27
STK36	18
STK39	100
SYK	100
TAK1	77
TAOK1	100
TAOK2	85
TAOK3	100
TBK1	100
TEC	9
TESK1	95
TGFBR1	60
TGFBR2	100
TIE1	25
TIE2	15
TLK1	93
TLK2	100
TNIK	100
TNK1	0
TNK2	1.1
TNNI3K	91
TRKA	16
TRKB	29

TRKC	45
TRPM6	100
TSSK1B	100
TSSK3	95
TTK	3.5
TXK	13
TYK2(JH1domain-catalytic)	100
TYK2(JH2domain-pseudokinase)	95
TYRO3	66
ULK1	99
ULK2	47
ULK3	84
VEGFR2	24
VPS34	63
VRK2	39

WEE1	100
WEE2	90
WNK1	73
WNK2	67
WNK3	46
WNK4	39
YANK1	30
YANK2	30
YANK3	100
YES	0
YSK1	84
YSK4	58
ZAK	13
ZAP70	100
